# Natural Variation in Vitamin B_1_ and Vitamin B_6_ Contents in Rice Germplasm

**DOI:** 10.3389/fpls.2022.856880

**Published:** 2022-04-04

**Authors:** Nathalie Mangel, Jared B. Fudge, Wilhelm Gruissem, Teresa B. Fitzpatrick, Hervé Vanderschuren

**Affiliations:** ^1^Plant Biotechnology, Department of Biology, ETH Zurich, Zurich, Switzerland; ^2^Vitamin & Environmental Stress Responses in Plants, Department of Botany and Plant Biology, Université de Genève, Geneva, Switzerland; ^3^Biotechnology Center, National Chung Hsing University, Taichung, Taiwan; ^4^Plant Genetics Laboratory, TERRA Teaching and Research Center, Gembloux Agro-Bio Tech, Université de Liège, Gembloux, Belgium

**Keywords:** rice, vitamin B_1_, vitamin B_6_, natural variation, germplasm, biofortification, micronutrient deficiency, hidden hunger

## Abstract

Insufficient dietary intake of micronutrients contributes to the onset of deficiencies termed hidden hunger—a global health problem affecting approximately 2 billion people. Vitamin B_1_ (thiamine) and vitamin B_6_ (pyridoxine) are essential micronutrients because of their roles as enzymatic cofactors in all organisms. Metabolic engineering attempts to biofortify rice endosperm—a poor source of several micronutrients leading to deficiencies when consumed monotonously—have led to only minimal improvements in vitamin B_1_ and B_6_ contents. To determine if rice germplasm could be exploited for biofortification of rice endosperm, we screened 59 genetically diverse accessions under greenhouse conditions for variation in vitamin B_1_ and vitamin B_6_ contents across three tissue types (leaves, unpolished and polished grain). Accessions from low, intermediate and high vitamin categories that had similar vitamin levels in two greenhouse experiments were chosen for in-depth vitamer profiling and selected biosynthesis gene expression analyses. Vitamin B_1_ and B_6_ contents in polished seeds varied almost 4-fold. Genes encoding select vitamin B_1_ and B_6_ biosynthesis *de novo* enzymes (*THIC* for vitamin B_1_, *PDX1.3a*–*c* and *PDX2* for vitamin B_6_) were differentially expressed in leaves across accessions contrasting in their respective vitamin contents. These expression levels did not correlate with leaf and unpolished seed vitamin contents, except for *THIC* expression in leaves that was positively correlated with total vitamin B_1_ contents in polished seeds. This study expands our knowledge of diversity in micronutrient traits in rice germplasm and provides insights into the expression of genes for vitamin B_1_ and B_6_ biosynthesis in rice.

## Introduction

Vitamin B_1_ (thiamine) and vitamin B_6_ (pyridoxine) are among the water-soluble vitamins that are essential micronutrients for humans and other animals. Several chemically related forms of vitamin B_1_ and B_6_ are found, termed vitamers, with thiamine diphosphate (TDP) and pyridoxal-5′-phosphate (PLP) the most well-known vitamers, respectively, owing to their known coenzyme functions in primary metabolism across all kingdoms (Colinas and Fitzpatrick, 2015). As humans obtain the bulk of vitamins from dietary sources, micronutrient deficiency disorders often arise when dietary intake is insufficient, either alone or in concert with congenital factors such as impaired vitamin metabolism and/or certain lifestyles (Bailey et al., 2015). Micronutrient deficiency (“hidden hunger”) is a global health problem, with over 2 billion people estimated to have one or more micronutrient deficiencies (Bailey et al., 2015). Monotonous consumption of staple crops with low dietary levels of vitamin B_1_ and B_6_ contributes to non-attainment of the recommended dietary allowance (RDA) of vitamin B_1_ (for adults: 1.1–1.3 mg/day) and B_6_ (for adults: 1.3–2.0 mg/day; Fitzpatrick et al., 2012; Titcomb and Tanumihardjo, 2019). Additional factors may further contribute to the inability to reach micronutrient RDAs, such as post-harvest deterioration, certain food processing and preparation practices, or consumption of foodstuffs containing vitamin-degrading enzymes (Van Der Straeten et al., 2020). Furthermore, the COVID-19 pandemic has reduced incomes in 63 low- and middle-income countries and increased the proportion of people unable to afford healthy diets, with implications for calorific and micronutrient deficiencies (Laborde et al., 2021).

Vitamin B_1_ deficiency disorders (also known as thiamine deficiency disorders) are endemic in several low- and middle-income countries in India, Asia, and Africa, in particular where polished rice is a major source of calories and interventions to improve dietary micronutrient intake are absent (Johnson et al., 2019; Whitfield et al., 2021). Deficiency in vitamin B_1_ may lead to a broad spectrum of neurological disorders including beriberi and tropical ataxia neuropathy (Whitfield et al., 2021), with Wernicke’s encephalopathy and Wernicke-Korsakoff syndrome prevalent in alcoholics (Isenberg-Grzeda et al., 2012; Latt and Dore, 2014; Dhir et al., 2019). Vitamin B_1_ deficiency also occurs in high income countries and may arise through diverse pathophysiological mechanisms beyond alcoholism (Gomes et al., 2021; Onishi et al., 2021). Congenital defects in vitamin B_1_ metabolism are also known to result in deficiency disorders (Ortigoza-Escobar et al., 2016). Although data at the population level is lacking for vitamin B_1_ deficiency compared to other micronutrients, studies on Chinese cohorts showed 91.8% of children and 81.7% of adults over 60 years do not reach their dietary estimated daily intake of vitamin B_1_ (Wang et al., 2017; Liu et al., 2019). Vitamin B_6_ deficiency may manifest with seizures and other neurological events, because of the role of PLP in neurotransmitter biosynthesis (Wilson et al., 2019; Akiyama et al., 2020; Rojo-Sebastián et al., 2020). Genetic defects in vitamin B_6_ metabolism could also lead to vitamin B_6_-dependent epilepsy (Wilson et al., 2019). Vitamin B_6_ deficiencies have been associated with low socioeconomic status (Liu et al., 2019; Zhu et al., 2020), certain medications (Lussana et al., 2003; Allen et al., 2015; Porter et al., 2019) and a range of diseases including inflammation (Paul et al., 2013), diabetes (Nix et al., 2015; Porter et al., 2019), cardiovascular disease (Dhalla et al., 2013; Wei and Ji, 2020), and certain cancers (Gylling et al., 2017). Lee (2021) reviews in detail vitamin B_6_ deficiency and women’s health issues. In countries where vitamin B_6_ status has been studied at the population level, 24% of Americans are at risk or deficient in vitamin B_6_ (Bird et al., 2017), rising to one-third in South Korea (Kim and Cho, 2014). Among a Chinese cohort, 95.1% of subjects aged 60 years and over were marginal or deficient for vitamin B_6_ status (Liu et al., 2019). Increased dietary intake of vitamin B_1_ and B_6_ is therefore likely to assist in mitigating respective deficiency disorders in a diverse range of populations (Titcomb and Tanumihardjo, 2019).

In addition to the well-established roles as enzymatic cofactors and the micronutrient deficiency disorders that may arise in turn when scarce, B_1_ and B_6_ vitamers are implicated in environmental stress responses in plants (Fitzpatrick, 2011; Fitzpatrick and Chapman, 2020). Both vitamins exhibit antioxidant activity *in vitro* (Gliszczyńska-Świgło, 2006; Czégény et al., 2019) and vitamin B_6_ can quench singlet oxygen in plants (Denslow et al., 2005) and fungi (Bilski et al., 2000). In cases where mutant alleles are non-lethal, downregulation or knock out of certain vitamin B_1_ and B_6_ metabolism genes typically results in stunting or increased susceptibility to various plant pathogens (Fudge et al., 2017; Fitzpatrick and Chapman, 2020). Protective effects of both vitamin B_1_ and B_6_ have been observed under abiotic stress, together with an induction of biosynthesis *de novo* (Ribeiro et al., 2005; Denslow et al., 2007; Rapala-Kozik et al., 2008; Tunc-Ozdemir et al., 2009; Rapala-Kozik et al., 2012; Huang et al., 2013; Moccand et al., 2014; Dell’Aglio et al., 2017). Exogenous vitamin B_1_ provision could induce expression of defense-related genes and “prime” plants for enhanced resistance upon subsequent pathogen challenge (Ahn et al., 2005, 2007; Boubakri et al., 2012; Huang et al., 2016). Mutants impaired in vitamin B_6_ metabolism are hyper-sensitive to abiotic stress and are susceptible to pathogen challenge (Vanderschuren et al., 2013; Zhang et al., 2015; Samsatly et al., 2020). Despite this body of evidence, relatively little is known about the molecular dialog between stress signaling and vitamin metabolism. Hanson et al. (2016) hypothesized that protective effects from exogenous vitamin supplementation complement acute cofactor deficiencies *in planta* upon stress, as opposed to simply having direct antioxidant properties. Taken together, crop varieties with high vitamin B_1_ and B_6_ contents may confer some resistance to stress through being poised to readily supply TDP and PLP to apoenzymes when demands on primary metabolism increase.

**Figure 1:**
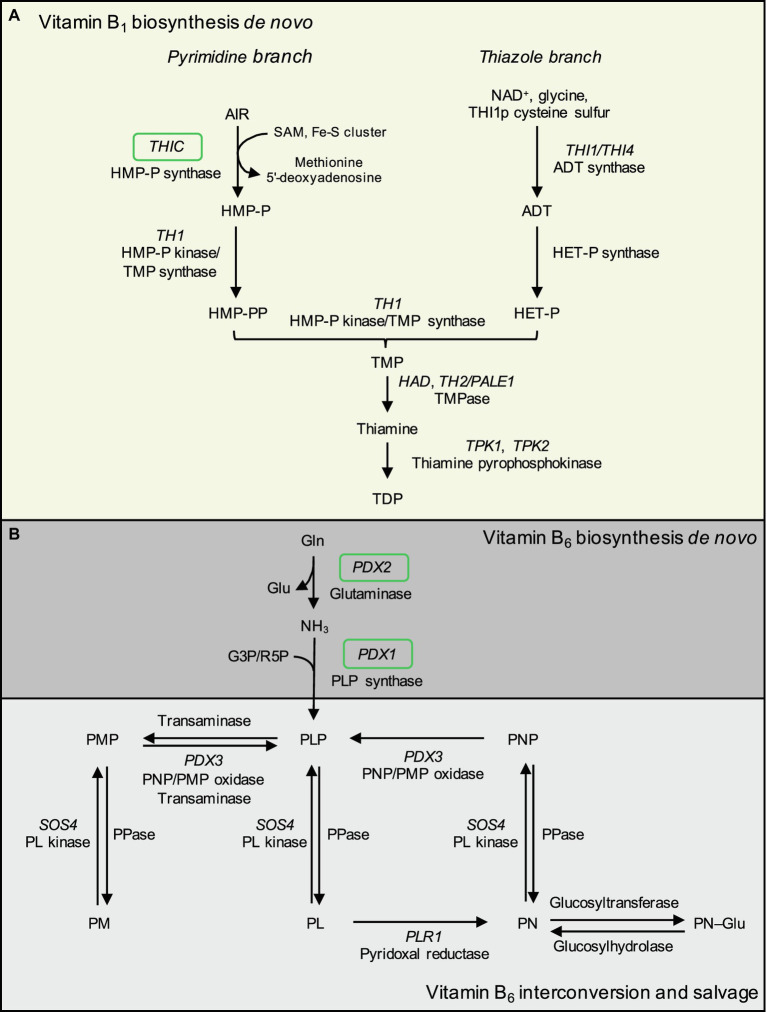
Biosynthesis *de novo* of vitamin B_1_ and B_6_ in plants based on Arabidopsis. **(A)** Vitamin B_1_ biosynthesis *de novo* is predominantly localized to the chloroplast and comprises formation of pyrimidine and thiazole heterocycles linked by a methylene bridge. The pyrimidine moiety of vitamin B_1_ is supplied *via* conversion of 5-amino-imidazole ribotide (AIR) into 4-amino-5-hydroxymethyl-2-pyrimidine phosphate (HMP-P) by HMP-P synthase (encoded by *THIC*) in an iron-sulfur cluster and SAM-dependent reaction, with the release of methionine and a 5′-deoxyadenosine radical. Next, HMP-P is phosphorylated to HMP-PP by the bifunctional HMP-P kinase/TMP synthase (encoded by *THIAMIN REQUIRING 1, TH1*). The thiazole moiety of vitamin B_1_ is produced from nicotinamide adenine dinucleotide (NAD^+^), glycine, and a sulfur atom donated from a conserved cysteine residue from the single-turnover enzyme ADT synthase/THI4 (encoded by *THI1* in plants, *THI4* in yeast) to produce adenosine diphosphate thiazole (ADT). ADT is converted to hydroxyethylthiazole phosphate (HET-P) by HET-P synthase activity (loci encoding this activity currently unconfirmed). HET-P and HMP-PP are condensed by TMP synthase activity of TH1 to form thiamine monophosphate (TMP). TMP is dephosphorylated by TMP phosphatases (TMPase) including TH2/PALE1 (THIAMIN REQUIRING 2/PALE GREEN 1) and HALOACID DEHALOGENASE (HAD), forming free thiamine. The cofactor vitamer thiamine diphosphate (TDP) is generated by diphosphorylation of free thiamine at the hydroxyl position by thiamine pyrophosphokinases (encoded by At*TPK1–2*, Os*TPK1–3*). **(B)** Vitamin B_6_ biosynthesis *de novo* is localized to the cytosol to form the cofactor vitamer pyridoxal 5′-phosphate (PLP). PDX2 is a glutaminase that yields glutamate and ammonia, the latter is used by PLP synthase (PDX1, encoded by *PDX1.1*–*1.3* in Arabidopsis and *PDX1.3a*–*c* in rice) to form PLP with the addition of either ribose 5-phosphate (R5P) or glyceraldehyde 3-phosphate (G3P). B_6_ vitamers are interconverted in a salvage pathway with known components localized to the cytosol, mitochondria, and chloroplasts. Unknown phosphatases convert phosphorylated vitamers to non-phosphorylated vitamers. Pyridoxal kinase (encoded by *SALT OVERLY SENSITIVE 4*, *SOS4*) phosphorylates pyridoxal (PL), pyridoxine (PN), and pyridoxamine (PM) at the 5′ position. PL is converted to PN by pyridoxal reductase (encoded by *PLR1*). The PNP/PMP oxidase (encoded by *PDX3*) converts pyridoxine 5′-phosphate (PNP) and pyridoxamine 5′-phosphate (PMP) to PLP. Unknown glucosyltransferases and glucosylhydrolases convert PN to PN–Glu and vice versa. At: *Arabidopsis thaliana*. Os: *Oryza sativa*. Green boxes denote genes assayed by qRT-PCR in this study.

Biosynthesis *de novo* of vitamin B_1_ and B_6_ in plants (Figure 1) has largely been unraveled in model species, chiefly Arabidopsis (*Arabidopsis thaliana*, At), maize (*Zea mays*, Zm), and tobacco (*Nicotiana tabacum*, Nt; Colinas and Fitzpatrick, 2015). This has permitted rational design of metabolic engineering strategies toward biofortifying staple crops with enhanced levels of these vitamins in consumed tissues (Fudge et al., 2017; Goyer, 2017; Strobbe and Van Der Straeten, 2018). However, several limitations have been reported, including marginal increases in vitamin contents through upregulation of biosynthetic pathways, with limited over-accumulation of vitamins in the target tissues and organs (Dong et al., 2015, 2016; Mangel et al., 2019; Strobbe et al., 2021a,b). Further effort is therefore required to overcome bottlenecks to biofortify vitamin B_1_ and B_6_ in rice endosperm at meaningful levels to approach respective RDAs.

Germplasm of certain crops has proven a useful source to biofortify maize, cassava, and sweet potato with provitamin A (ß-carotene; Bouis and Saltzman, 2017; Bechoff et al., 2018; Govender et al., 2019; Foley et al., 2021). Several crop species including cassava, potato, quinoa, rice, maize, wheat, and pulses have been studied for variation in B complex vitamins (Villareal and Juliano, 1989; Sotelo et al., 1990; Kennedy and Burlingame, 2003; Goyer and Navarre, 2007; Dong et al., 2011, 2014; Goyer and Haynes, 2011; Goyer and Sweek, 2011; Shewry et al., 2011; Robinson et al., 2015; Nowak et al., 2016; Singh et al., 2016; Mangel et al., 2017; Zarei et al., 2017; Bali et al., 2018; Freitag et al., 2018; Granda et al., 2018; Li et al., 2018; Guo et al., 2019; Riaz et al., 2019; Robinson et al., 2019; Jha et al., 2020). However, only some of these studies have sought to understand the genetic basis underpinning such variation and/or carried out detailed vitamer profiling and quantification by analytical techniques such as HPLC. Knowledge of constituent vitamer profiles and molecular determinants for high vitamin content accessions could aid the introgression of desirable alleles into farmer-preferred crop varieties to deliver bioavailable vitamers to consumers most in need. Such detailed knowledge of rice germplasm is limited with respect to vitamin B_1_ (Villareal and Juliano, 1989; Sotelo et al., 1990; Kennedy and Burlingame, 2003), while vitamin B_6_ contents are known only for three rice varieties from the United States (Zarei et al., 2017). No information on the molecular mechanisms behind such variation in rice have been published to our knowledge.

Rice is a staple crop for 50% of the world’s population, including some of the world’s poorest (Bhullar and Gruissem, 2013). Cooked white, polished rice (lacking the seed coat and embryo) is a poor source of several micronutrients, including both vitamin B_1_ and B_6_, when considered as the source of 80% of the daily calorific intake (Fitzpatrick et al., 2012; de Pee, 2014). As much as 90–98% of grain vitamin B_1_ contents are lost during polishing (Dong et al., 2016; Strobbe et al., 2021a) and up to 85% of vitamin B_6_ (Mangel et al., 2019). Therefore, rice endosperm is a rational target for improvement of vitamin B_1_ and B_6_ contents. Toward obtaining the upper ranges of the respective RDA (for lactating women) from a 233 g serving of cooked polished rice, 32- and 13.6-fold increases in vitamins B_1_ and B_6_ are required in polished seeds (Fudge et al., 2017).

Here, toward determining if rice germplasm could be exploited for endosperm biofortification with vitamin B_1_ and B_6_, we performed an in-depth characterization of a genetically diverse panel of 59 rice accessions grown under greenhouse conditions. The selected accessions spanning three subspecies were sourced from geographically diverse countries and vary in their resistance and susceptibility to major rice diseases. Accessions with contrasting vitamin contents in three tissue types were selected for in-depth vitamer profiling by HPLC. RT-qPCR assays of selected vitamin biosynthesis *de novo* genes revealed differential expression across the accessions, which did not correlate with vitamin contents in leaves. Accessions profiled here with the highest polished seed vitamin B_1_ and B_6_ contents did not display sufficient levels of vitamin B_1_ and B_6_ to meet the respective RDAs. Future efforts ought to consider the use of substantially larger germplasm panels, alongside additional metabolic engineering strategies, toward combatting vitamin B_1_ and B_6_ deficiencies for rice consumers in greatest need.

## Materials and Methods

### Plant Material, Growth, and Sampling Conditions

Rice accessions (Supplementary Table S1) were obtained as dry seeds from the International Rice Research Institute, Philippines, and selected using the International Rice GeneBank Collection Information System.^1^ Dry seeds were dehusked and surface sterilized in 70% (v/v) ethanol for 30 s, followed by 30 min of agitation in 1.5% (v/v) sodium hypochlorite solution +0.01% (v/v) Tween-20. Seeds were then rinsed five times in sterile water and then transferred to sterile plastic jars containing full-strength MS media (Murashige and Skoog, 1962) supplemented with 3% (w/v) sucrose and 0.3% (w/v) Gelrite, at pH 5.8. Seeds were incubated in darkness at 28°C for 48 h, before transfer to a growth cabinet for 12 d under 16 h light and 8 h darkness at 28°C. Seedlings were then transferred to a greenhouse under controlled conditions (12 h artificial light at 30°C and 80% humidity, 12 h darkness at 22°C, and 60% humidity). Three seedlings of the same accession were planted in one pot (pot diameter 18 cm, 12 cm height).

For experiment 1, 49 accessions were propagated simultaneously in growth cabinets prior to transfer of the seedlings to a greenhouse (June–August 2013, Eschikon, Switzerland). In a second independent experiment (hereinafter called experiment 2), 21 selected accessions grown in experiment 1 were propagated for a second time under greenhouse conditions (August 2014–October 2014, Eschikon, Switzerland), together with 10 additional accessions from the Oryza SNP Sequencing Project (McNally et al., 2009), resulting in 31 accessions grown in experiment 2 and a total of 59 accessions screened across both experiments. Sampling was consistently performed in the morning (10.30 am–12 pm). Leaves were sampled from three different tillers of 50 days old vegetative plants in experiment 1 and 40 days old plants in experiment 2. Leaf tissue was pooled, snap frozen in liquid nitrogen, and stored at −80°C until further use. Fully ripened panicles (maturing at different times owing to variation in heading dates) were harvested and dried for 5 d at 37°C. Dried, mature seeds were dehusked (referred to hereinafter as unpolished seeds) and stored at −80°C until further use, or polished for 2 min in a PEARLEST polisher (Kett) to remove the embryo, aleurone layer, and seed coat (polished seeds), and stored at −80°C until further use.

### Vitamin B_1_ and B_6_ Quantification

#### Vitamin B_1_

For vitamin B_1_, a *Saccharomyces cerevisiae thi4* mutant deficient in vitamin B_1_ biosynthesis *de novo* was used for quantification of total vitamin B_1_ from rice tissues from plants grown in experiments 1 and 2 (Raschke et al., 2007). Fifty milligrams of frozen, ground rice leaves or seeds were used for extraction of vitamin B_1_ in 500 μl of 20 mM sulfuric acid in darkness at room temperature for 30 min, before heating to 100°C for 1 h. The solution was adjusted to pH 5.7 with 3 M sodium acetate and centrifuged. To convert phosphorylated B_1_ vitamers to free thiamine to permit uptake by yeast, supernatants were treated with acid phosphatase (0.2 U/10 μl per 50 μl of plant extract) overnight for 12–15 h at 37°C. Total vitamin B_1_ was calculated from the linear range of a standard curve prepared with 5–100 ng of thiamine hydrochloride provided to the *thi4* yeast mutant in thiamine-deficient media.

Samples from candidate accessions with contrasting total vitamin B_1_ contents were selected for confirmation and vitamer profiling of thiochrome derivatives by HPLC using a method first described by Moulin et al. (2013). Fifty milligrams of frozen, ground rice leaves or seeds were used for extraction of soluble vitamin B_1_ in 100 μl in 1% (v/v) trichloroacetic acid by aggressive vortexing at room temperature for 30 min. Samples were centrifuged at full speed in a tabletop microcentrifuge for 10 min at room temperature. The clear supernatant was neutralized with 3 M sodium acetate to 10% of the final volume and oxidized to thiochrome derivatives using 15 μl of freshly prepared 30 mM potassium ferracyanide in 15% (w/v) NaOH, with 15 μl 1 M NaOH and 25 μl methanol according to Moulin et al. (2013). Samples were injected into an Agilent Technologies 1260 HPLC to determine vitamer profiles by separation of thiochrome derivatives on a Cosmosil *π*-NAP column (150 × 4.6 mm, 3 μm pore size) using a methanol gradient at 1 ml min^−1^ detailed in Moulin et al. (2013), with a 40 min run time. Peaks of fluorescence corresponding to the retention time of the commercial standards of B_1_ vitamers TDP (Sigma), thiamine monophosphate (TMP; Fluka), and thiamine (Fluka) were integrated and extrapolated against a standard curve for each vitamer. Peak area was integrated only from non-saturated peaks and in cases of peak saturation, samples were reinjected in lower volumes. Injection volumes ranged from 10 to 40 μl.

Data for leaf samples were normalized to fresh weight (FW) and seed samples to dry weight (DW).

#### Vitamin B_6_

For vitamin B_6_, a *Saccharomyces pastorianus* American Type Culture Collection 9080 strain was used (Tambasco-Studart et al., 2005) as reported (Mangel et al., 2019). Fifty milligrams of frozen, ground rice leaves or seeds were used for extraction of vitamin B_6_ in 500 μl of 20 mM sulfuric acid in darkness at room temperature for 30 min, before heating to 100°C for 1 h. The solution was adjusted to pH 5.7 with 3 M sodium acetate and centrifuged. To convert phosphorylated and glucosylated B_6_ vitamers to non-phosphorylated vitamers to permit uptake by yeast, supernatants were treated with acid phosphatase and β-glucosidase (0.2 U/10 μl of each enzyme per 50 μl of plant extract) overnight for 12–15 h at 37°C. Total vitamin B_6_ was calculated from the linear range of a standard curve prepared with 0.15–2.4 ng of pyridoxine hydrochloride provided to the yeast mutant in pyridoxine-deficient media.

Samples from candidate rice accessions with contrasting total vitamin B_6_ contents were selected for vitamer profiling by HPLC using an established protocol (Szydlowski et al., 2013). Fifty milligrams of frozen, ground rice leaves or seeds were used for extraction of vitamin B_6_ in 100 μl of 50 mM ammonium acetate pH 4.0 with aggressive vortexing for 10 min at room temperature. Samples were centrifuged at full speed in a tabletop microcentrifuge for 15 min at room temperature. The supernatant was incubated for 3 min at 99°C and again centrifuged for 15 min at room temperature before analysis. Extracts were injected into an Agilent Technologies 1200 HPLC to separate B_6_ vitamers on a Sunfire C18 column (Waters), 4.6 × 150 mm, 3.5 μm particle diameter, with post-column derivatization in 0.7 M potassium phosphate buffer with 1 g L^−1^ sodium bisulfite added freshly, flow rate 0.3 ml min^−1^. Samples were separated on an isocratic gradient of 50 mM ammonium acetate pH 4.0, flow rate 1 ml min^−1^ in a 40 min run time. Quantification was carried out using the linear range of a standard curve constructed with known amounts of standards (Colinas et al., 2016), with vitamin B_6_ glucoside (PN–Glu) determination calculated as PN equivalents (Mangel et al., 2019). Standards were prepared and injected into the HPLC with every set of extractions. Peak area was integrated only from non-saturated peaks and in cases of peak saturation, samples were reinjected in lower volumes. Injection volumes were typically 10–30 μl.

Data for leaf samples were normalized to fresh weight (FW) and seed samples to dry weight (DW).

### RNA Isolation and Gene Expression Analyses

RNA was isolated from rice leaf samples using an established protocol (Chang et al., 1993) with Mangel et al. (2019). Frozen, homogenized tissue was mixed with 1 ml of extraction buffer (2% w/v polyvinylpyrrolidone K-30, 100 mM Tris-HCl pH 8.0, 25 mM ethylenediaminetetraacetic acid, 2 M NaCl, and 0.5 g L^−1^ spermidine) and 2% (v/v) β-mercaptoethanol added freshly. Samples were incubated at 50°C for 15 min with agitation at 400–500 rpm, before being extracted twice with 1 volume of chloroform:isoamylalcohol (24:1, pH 7.5–8.0). Nucleic acids were recovered from the aqueous phase by absolute ethanol precipitation for 30 min at −80°C followed by centrifugation at top speed in a microcentrifuge for 30 min at 4°C. The pellet was washed in 80% (v/v) ethanol and resuspended in diethyl pyrocarbonate (DEPC)-treated water. RNA was precipitated overnight at −20°C in 2 M lithium chloride and collected by centrifugation at top speed in a microcentrifuge for 30 min at 4°C. The RNA pellet was washed sequentially in 80% (v/v) and absolute ethanol, before vacuum drying and resuspension in DEPC-treated water. RNA was quantified spectrophotometrically (Nanodrop) and stored at −80°C until further use. Expression of selected genes encoding vitamin B_1_ and B_6_ biosynthesis *de novo* enzymes was assayed by RT-qPCR. Two micrograms of total RNA were converted to cDNA with random hexamer oligonucleotide primers using the RevertAid First Strand cDNA Synthesis kit (Thermo Fisher) in accordance with the manufacturer’s instructions. Real-time qPCR reactions were prepared in 10 μl reaction volumes comprising 5 μl of 2x Fast SYBR Green master mix (Applied Biosystems), 2 μl of template cDNA diluted 5-fold in DEPC-treated water, 1 μl each of forward and reverse primer (1 μM working concentration), and 1 μl DEPC-treated water. A Roche LightCycler 480 II real-time qPCR machine was used with an initial denaturation at 95°C for 2 min, followed by 40 cycles of denaturation at 95°C for 10 s, annealing at 60°C for 20 s, and extension at 72°C for 30 s. Target gene expression was normalized to the Os*UBQ5* reference gene (Jain et al., 2006) and relative expression calculated using the delta-delta C_t_ method (Livak and Schmittgen, 2001). Samples for RT-qPCR were pipetted in duplicate and amplicon identity confirmed by melt curve analysis and Sanger sequencing. Primers for RT-qPCR were developed using Nipponbare genome sequences obtained from Phytozome v7_JGI (Goodstein et al., 2012). Sequences of oligonucleotide primers used for RT-qPCR are listed in Supplementary Table S2. Conservation of oligonucleotide primer binding sites in sequenced *Oryza sativa* accessions could be confirmed for Nipponbare (*japonica*), IR64 (*indica*), and I-Kung-Pao (*indica*), by search of the rice Molecular Breeding Knowledgebase.^2^ Multiple alignments were assembled for alleles belonging to these three accessions and the number of accessions from each subspecies belonging to a given GID group (i.e., bearing a given allele) are shown in Supplementary Figure S1. qRT-PCR threshold cycle (C_t_) values for the reference gene *UBQ5* are shown in Supplementary Table S3.

### Statistical Analyses

Data were analyzed in GraphPad Prism (version 9) and R (R Core Team, 2013). To determine the effect of accession on vitamin contents and leaf biosynthetic gene expression levels, one-way ANOVA tests were carried out with post-hoc Tukey tests ( α = 0.05) to correct for multiple comparisons. Statistically significant differences between accessions are denoted by different letters in respective figures and tables. For correlation analyses of total vitamin contents with leaf biosynthetic gene expression levels, Pearson’s correlation coefficients were determined in GraphPad Prism with two-tailed value of *p* tests and 95% confidence intervals. Standard deviations of ratios were estimated using the Taylor expansion formula.

## Results

### Vitamin B_1_ and B_6_ Contents in 59 Genetically Diverse Rice Accessions

**Figure 2:**
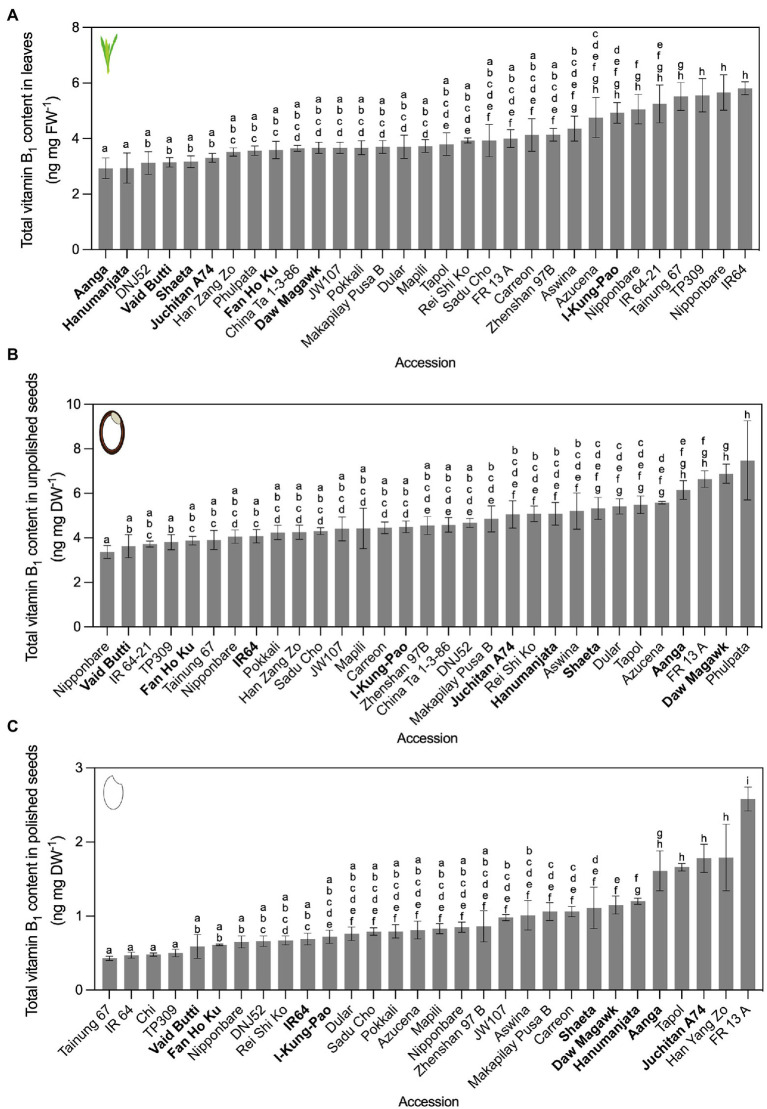
Vitamin B_1_ contents quantified by yeast assay. Leaves **(A)**, unpolished seeds **(B)**, and polished seeds **(C)** in rice accessions grown under greenhouse conditions and quantified by yeast assay. Twenty-one accessions cultivated in experiment 1 were re-sown, alongside 10 additional accessions from the Oryza SNP Project. The accessions with vitamin B_1_ content below the 25th percentile of the distribution were considered as low vitamin B_1_ accessions and those with vitamin content above the 75th percentile were considered as high vitamin B_1_ accessions. Low, intermediate, and high vitamin B_1_ accessions selected for HPLC analysis are in bold. Data are mean ± SD of 3 biological replicates, except Nipponbare (*n* = 6), IR64 (*n* = 6) and TP309 (*n* = 6) for the three tissues; IR64-21 (*n* = 2) for leaves; Nipponbare and IR-64-21 are *n* = 2 for unpolished seeds; and Nipponbare (*n* = 2), IR64-21 (*n* = 2) and Phulpata (*n* = 0) for polished seeds. The effect of accession on total vitamin B_1_ contents in panels **(A–C)** was determined by one-way ANOVA (*α* = 0.05) with multiple comparisons and Tukey’s *post-hoc* test. Statistically significant differences between accessions are denoted by different letters.

**Figure 3:**
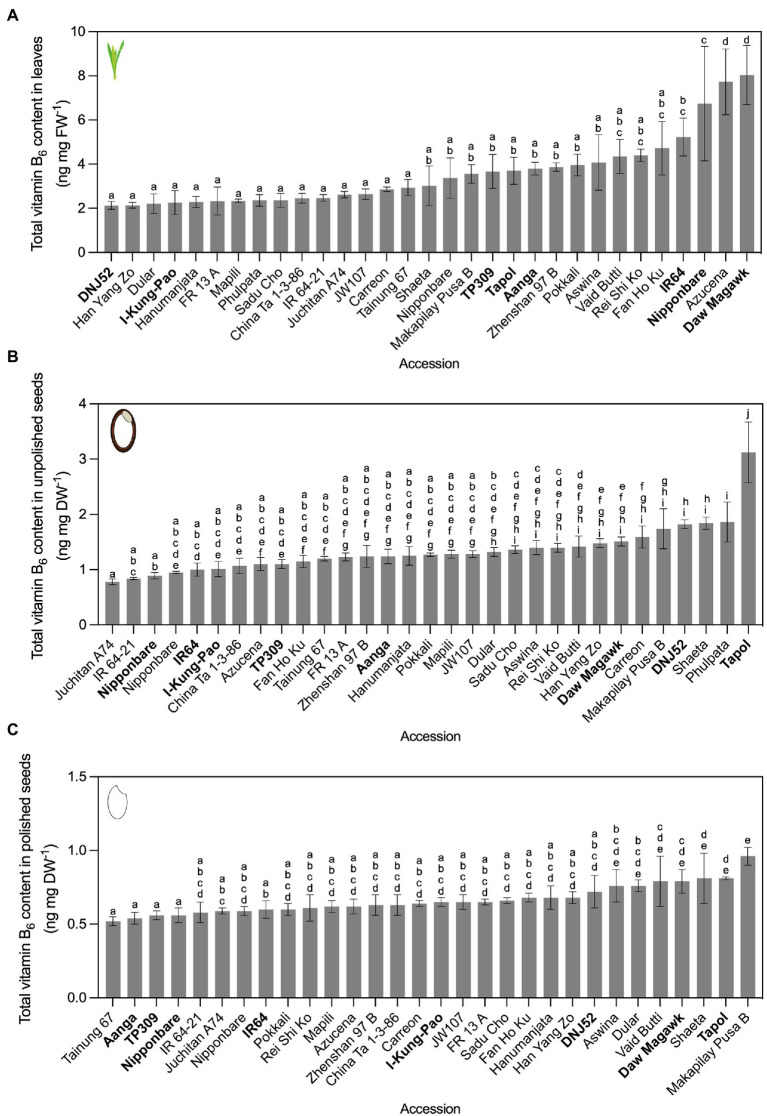
Vitamin B_6_ contents quantified by yeast assay. Leaves **(A)**, unpolished seeds **(B)**, and polished seeds **(C)** in contrasting rice accessions grown under greenhouse conditions and quantified by yeast assay. Twenty-one accessions cultivated in experiment 1 were re-sown, alongside 10 additional accessions from the Oryza SNP Project (experiment 2). The accessions with vitamin B_6_ content below the 25th percentile of the distribution were considered as low vitamin B_6_ accessions and those with vitamin content above the 75th percentile were considered as high vitamin B_6_ accessions. Low, intermediate, and high vitamin B_6_ accessions selected for HPLC analysis are bolded. Mean ± SD of 3 biological replicates, except Nipponbare (*n* = 6), IR64 (*n* = 6), and TP309 (*n* = 6) for the three tissues; IR 64–21 (*n* = 2) for leaves; Nipponbare (*n* = 2) and IR 64–21 (*n* = 2) for unpolished seeds; and Nipponbare (*n* = 2), IR 64–21 (*n* = 2) and Phulpata (*n* = 0) in polished seeds. The effect of accession on total vitamin B_6_ contents in panels **(A–C)** was determined by one-way ANOVA (*α* = 0.05) with multiple comparisons and Tukey’s *post-hoc* test. Statistically significant differences between accessions are denoted by different letters.

To obtain insights into the diversity of vitamin B_1_ and B_6_ contents in rice germplasm, we selected a total of 59 accessions for analysis from geographically diverse countries (Supplementary Table S1). Selected accessions included landraces and modern breeding lines spanning *O. sativa* subspecies *japonica*, *indica*, and *javanica*, ten accessions characterized in the OryzaSNP project (McNally et al., 2009), and with contrasting susceptibility and resistance to major rice diseases. These accessions were cultivated under greenhouse conditions across two independent plantings (experiment 1 and 2). To control for seasonal differences in greenhouse cultivation between the two independent plantings, plants were phenotyped in each experiment. The range of measured variation for accessions cultivated twice remained largely similar for plant height, number of panicles, and leaf dry weight (Supplementary Table S4). Total vitamin B_1_ and B_6_ contents in leaves, unpolished seeds, and polished seeds were first quantified using a yeast assay (Tambasco-Studart et al., 2005; Raschke et al., 2007; Figures 2, 3; Supplementary Tables S5, S6). Statistically significant differences in vitamin contents between accessions were determined in all cases by one-way ANOVA with multiple comparisons and Tukey’s *post-hoc* tests (*α* = 0.05). Accessions were placed into low, intermediate or high vitamin content groups based on their ranking below the 25th percentile, between the 25th and 75th percentile, or above the 75th percentile, respectively. Groups below the 25th percentile and above the 75th percentile were referred to as contrasting groups.

In experiment 1, 49 accessions were cultivated, with leaf tissues harvested from vegetative plants 50 days after germination (Supplementary Table S5A). Leaf vitamin B_1_ contents varied 3.32-fold, with Hanumanjata at 1.11 ng mg FW^−1^ as the lowest and Tapol with 3.68 ng mg FW^−1^ as the highest. Unpolished seed vitamin B_1_ contents varied 3.9-fold, from 2.35 ng mg DW^−1^ for Vaid Butti to Phulpata at 9.15 (± 1.11) ng mg DW^−1^ (Supplementary Table S5B). This was similar to previously reported ranges when converted to ng mg DW^−1^ (Villareal and Juliano, 1989; Sotelo et al., 1990; Kennedy and Burlingame, 2003), serving as benchmarks for six accessions assayed both here and previously. Polished seed vitamin B_1_ contents varied 2.72-fold, from 0.65 ng mg DW^−1^ for Hsinchu 56 to Hang Yang Zo at 1.76 ng mg DW^−1^ (Supplementary Table S5C). Twenty-one accessions across the three percentile groups from experiment 1 were cultivated a second time under the same greenhouse conditions for experiment 2, together with 10 additional accessions from the Oryza SNP Project (McNally et al., 2009). For vitamin B_1_ contents in experiment 2, leaf samples varied 1.98-fold, ranging from 2.93 ng mg FW^−1^ in Aanga to 5.81 ng mg FW^−1^ in IR64 (Figure 2A). Unpolished seed vitamin B_1_ contents varied 2.23-fold, from 3.36 ng mg DW^−1^ for Nipponbare to Phulpata at 7.48 ng mg DW^−1^ (Figure 2B). Polished seed vitamin B_1_ contents varied 6.06-fold, from 0.43 ng mg DW^−1^ for Tainung 67 to FR 13 A at 2.58 ng mg DW^−1^ (Figure 2C). Seed polishing led to a reduction in vitamin B_1_ content ranging between 57.9 and 89.3% (Supplementary Table S6). Although vitamin B_1_ contents showed some variation between experiment 1 and 2, the ranking of certain accessions based on vitamin B_1_ contents was not significantly different between the two experiments (Figure 2; Supplementary Table S5). Based on vitamin B_1_ contents, nine accessions from experiment 2 were selected for confirmation and in-depth vitamer profiling by HPLC and biosynthetic gene expression assays (see below).

Variation in vitamin B_6_ contents in rice germplasm was probed in an identical screening strategy as for vitamin B_1_. In experiment 1, vitamin B_6_ in leaves varied 4.64-fold, from 0.88 ng mg FW^−1^ in Hanumanjata to 4.07 ng mg FW^−1^ in Indane (Supplementary Table S7A). Unpolished seed vitamin B_6_ contents varied 3.9-fold, from 0.75 ng mg DW^−1^ for IR64 to Tapol at 2.49 ng mg DW^−1^ (Supplementary Table S7B). Polished seed vitamin B_6_ contents varied 2.94-fold, from 0.34 ng mg DW^−1^ for Fan Ho Ku to Phulpata at 1.01 ng mg DW^−1^ (Supplementary Table S7C). In experiment 2, vitamin B_6_ in leaves varied 3.77-fold, from 2.13 ng mg FW^−1^ in DNJ52 to 8.04 ng mg FW^−1^ in Daw Magawk (Figure 3A), with higher ranges compared to experiment 1. Seed samples were more stable in terms of variation between experiments compared to leaves, with unpolished seed vitamin B_6_ contents varying 4.0-fold, from 0.75 ng mg DW^−1^ for IR64 to Tapol at 3.12 ng mg DW^−1^ (Figure 3B). Consistent with our previous report (Mangel et al., 2019), polishing of rice seeds (that is, removal of the seed coat, embryo, and aleurone) results in losses of vitamin B_6_ contents in seeds for dietary intake, with a reduction in vitamin B_6_ content between unpolished and polished seeds ranging from 20% (Juchitan A74) to 76.5% (Aanga; Figure 3C; Supplementary Table S8). Similar to the vitamin B_1_ greenhouse screen results, vitamin B_6_ contents and fold changes also showed some variation between experiments. Several accessions from contrasting groups maintained similar vitamin B_6_ contents and eight were selected from experiment 2 for confirmation and vitamer profiling by HPLC and biosynthetic gene expression assays (see below).

### B_1_ and B_6_ Vitamer Profiling by HPLC of Three Tissues in Contrasting Rice Accessions

**Figure 4:**
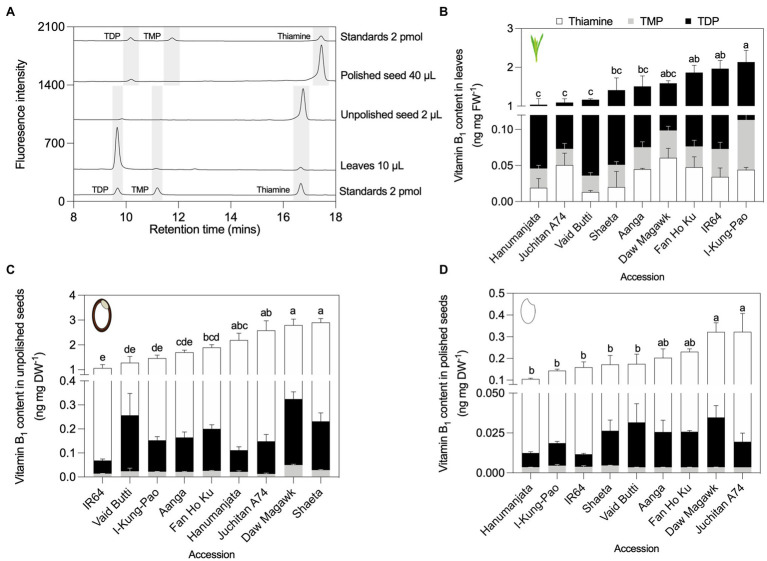
HPLC profiling of B_1_ vitamers in leaves and seeds of rice accessions contrasting in total vitamin B_1_ contents. **(A)** Representative HPLC chromatograms of leaf and seed tissues. To facilitate visualization, chromatograms were offset from the baseline by 50 units for standards at bottom of the panel, 350 units for leaves, 950 units for unpolished seeds, 1,400 units for polished seeds, and 1,900 units for standards used for quantification of polished seed samples. Shifts in retention time can occur as a result of slight changes to the pH of HPLC running buffers between experiments. HPLC analysis of B_1_ vitamers in leaves **(B)**, unpolished seeds **(C)**, and polished seeds **(D)**. Samples were obtained from plant material grown under greenhouse conditions in experiment 2. Leaves were sampled from 40-day-old plants. Seed samples were obtained from fully mature pannicles and dried before extraction. Data are the mean ± standard deviation of *n* = 3 biological replicates. The effect of accession on total vitamin B_1_ contents in panels **(B–D)** was determined by one-way ANOVA (*α* = 0.05) with multiple comparisons and Tukey’s *post-hoc* test. Statistically significant differences between accessions are denoted by different letters.

To validate the yeast assay screen data for respective total vitamin contents in three rice tissue types from selected accessions with low, intermediate and high vitamin contents, a precise quantification of their soluble constituent vitamers was performed by HPLC against known amounts of external standards (Moulin et al., 2013; Szydlowski et al., 2013). For B_1_ vitamers, profiles were obtained for TDP, TMP, and thiamine. B_6_ vitamers quantified were pyridoxal (PL), pyridoxamine (PM), pyridoxine (PN), and their phosphorylated esters. Since as much as 50% of vitamin B_6_ is found as glucosides in certain plant samples (Gregory, 1998), pyridoxine glucosides (PN–Glu) were determined as “PN equivalents” by treatment of samples with a ß-glucosidase and the increase in PN interpreted as PN–Glu (Mangel et al., 2019). HPLC assays revealed that the most abundant B_1_ vitamer in rice leaf tissues was TDP (94–97%), followed by minor but detectable pools of TMP and thiamine (Figures 4A,B; Supplementary Table S9A). This is consistent with previous observations in wild-type rice leaf samples (Dong et al., 2016; Hsieh et al., 2021), maize (Guan et al., 2014), Arabidopsis (Mimura et al., 2016; Hofmann et al., 2020), and cassava (Mangel et al., 2017). Total vitamin B_1_ (the sum of TDP, TMP, and thiamine) in the leaves of rice accessions assayed here showed statistically significant 2.07-fold variation, ranging from 1.04 in Hanumanjata to 2.13 ng mg FW^−1^ in I-Kung-Pao [*F*(6, 14) = 7.331, *p* = 0.0011, Figure 4B; Supplementary Table S9A]. In contrast to leaf samples, vitamin B_1_ contents in unpolished and polished rice seeds largely comprised free thiamine (80–95%), with low amounts of TDP and trace levels of TMP (Figures 4A,C,D; Supplementary Tables S9B,C). Unpolished seed samples varied 2.72-fold, ranging from 1.07 in IR64 to 2.90 ng mg DW^−1^ in Shaeta [*F*(8, 18) = 21.41, *p* < 0.0001; Figure 4C; Supplementary Table S9B]. As the seed coat, embryo, and aleurone cell layer are the rice seed compartments that store the bulk of vitamin B_1_, polishing rice grain is known to deplete grain thiamine contents by as much as 90–98% (Dong et al., 2016; Strobbe et al., 2021a). Here, total vitamin B_1_ in polished seed samples decreased approximately 10-fold compared to unpolished seeds, with 3.94-fold variation between the lowest and highest accessions, ranging from 0.1 in Hanumanjata to 0.3 ng mg DW^−1^ in Juchitan A74 [*F*(8, 18) = 8.3, *p* = 0.0001; Figure 4D; Supplementary Table S9C].

**Figure 5:**
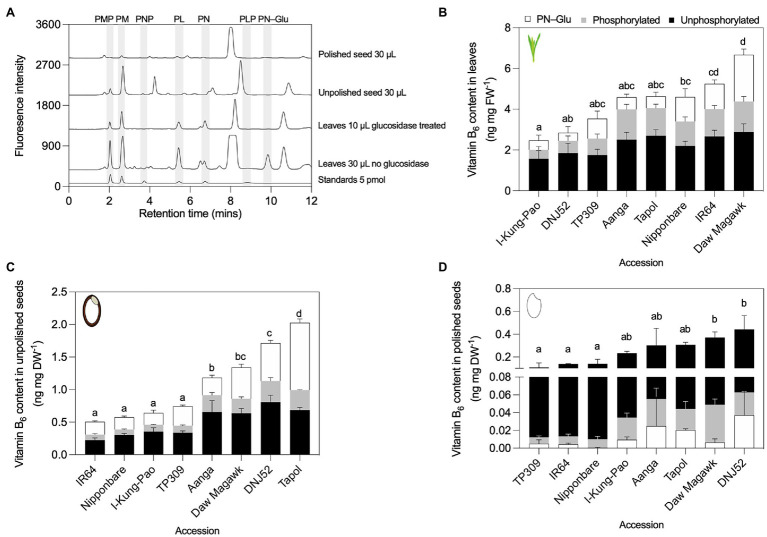
HPLC profiling of B_6_ vitamers in leaves and seeds of rice accessions contrasting in total vitamin B_6_ contents. **(A)** Representative HPLC chromatograms of leaf and seed tissues. To facilitate visualization, chromatograms were offset from the baseline of the standards by 300 units for leaves 30 μl injected no glucosidase, 1,200 units for leaves 10 μl injected glucosidase treated, 2,000 units for unpolished seed, and 2,700 units for polished seed. HPLC analysis of B_6_ vitamers in leaves **(B)**, unpolished seeds **(C)**, and polished seeds **(D)**. Samples were obtained from plant material grown under greenhouse conditions in experiment 2. Leaves were sampled from 40-day-old plants. Seed samples were obtained from fully mature pannicles and dried before extraction. Data are the mean ± standard deviation of *n* = 3 biological replicates. The effect of accession on total vitamin B_6_ contents in panels **(B–D)** was determined by one-way ANOVA (*α* = 0.05) with multiple comparisons and Tukey’s *post-hoc* test. Statistically significant differences between accessions are denoted by different letters.

In contrasting accessions selected for HPLC analysis of vitamin B_6_ contents, leaf samples varied 2.24-fold between the selected accessions, from 2.44 in I-Kung-Pao to 5.48 ng mg FW^−1^ in Daw Magawk [*F*(7, 16) = 12.91, *p* < 0.001; Figures 5A,B; Supplementary Table S10]. Unphosphorylated vitamers (PL, PM, and PN) comprised the majority of vitamers in leaves of all accessions, with relative proportions of PN–Glu increasing in accessions with higher total vitamin B_6_ (Figure 5B; Supplementary Table S10A). Unphosphorylated B_6_ vitamers similarly comprised the majority of the vitamin B_6_ contents of Arabidopsis shoot material and is consistent with a previous observation for leaves of wild-type TP309 rice (Raschke et al., 2011; Mangel et al., 2019). For unpolished seeds, the total vitamin B_6_ contents varied 3.93-fold between the lowest and highest accessions, with 0.45 in IR64 to 1.78 ng mg DW^−1^ in Tapol [*F*(7, 16) = 65.63, *p* < 0.001; Figures 5A,C; Supplementary Table S10B]. In general, unphosphorylated B_6_ vitamers and PN–Glu comprised the bulk of vitamers in unpolished seeds (Figure 5C; Supplementary Table S9B). Similar to vitamin B_1_, polishing of rice grains depletes vitamin B_6_ stores by as much as 85% (Mangel et al., 2019). Polished seed vitamin B_6_ contents varied 3.95-fold between the lowest and highest accessions, corresponding to TP309 at 0.1 and 0.41 ng mg DW^−1^ for DNJ52 [*F*(7, 16) = 3.918, *p* = 0.0113]. Unphosphorylated vitamers comprised the bulk of polished seed vitamin B_6_ pools in all accessions, while phosphorylated vitamers (PLP, PMP, and PNP) and PN–Glu comprised relatively minor constituents or were below the detection limit (Figures 5A,D; Supplementary Table S10C).

### Vitamin B_1_ and B_6_ Biosynthesis *de novo* Gene Expression in Contrasting Accessions

**Figure 6:**
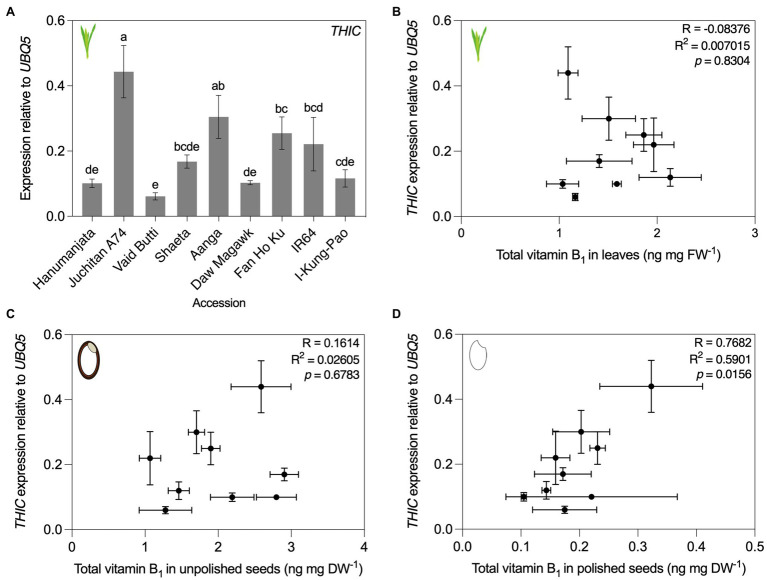
*THIC* expression in leaves and correlation of accessions with contrasting vitamin B_1_ contents. **(A)** RNA was isolated from plant material used in Figure 4B for qRT-PCR assay of *THIC* expression. Expression was normalized to *UBQ5* and data are the mean ± standard deviation of *n* = 3 biological replicates. The effect of accession on gene expression was determined by one-way ANOVA (*α* = 0.05) with multiple comparisons and Tukey’s *post-hoc* test. Statistically significant differences between accessions are denoted by different letters. Accessions are ordered according to leaf total vitamin B_1_ content as determined in Figure 4B. **(B–D)** Correlation of *THIC* gene expression from panel **(A)** with leaf **(B)** total vitamin B_1_ contents in Figure 4B by Pearson’s correlation coefficient test (*p* < 0.05), unpolished seed from Figure 4C **(C)**, and polished seed total vitamin B_1_ contents from Figure 4D **(D)**.

**Figure 7:**
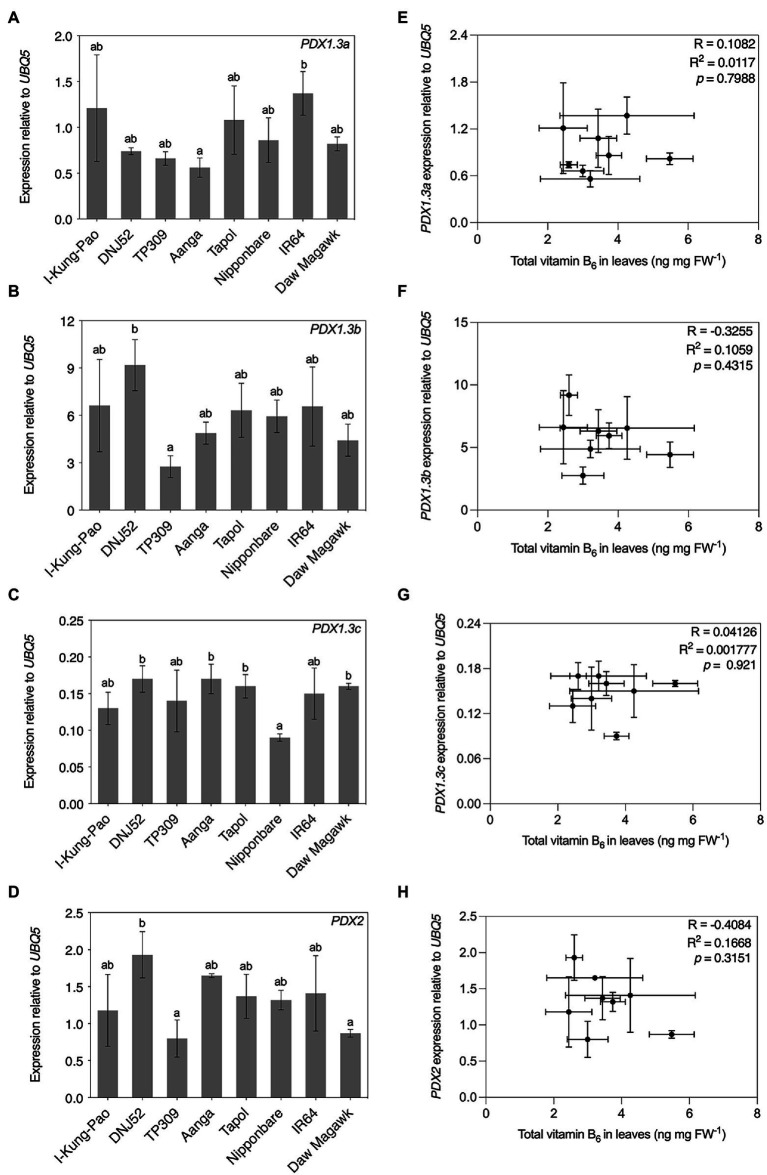
Expression and correlation of select genes for vitamin B_6_ biosynthesis *de novo* enzymes in leaves of accessions with contrasting vitamin B_6_ contents. RNA was isolated from plant material used in Figure 5B for qRT-PCR assays of *PDX1.3a* **(A)**, *PDX1.3b* **(B)**, *PDX1.3c* **(C)**, and *PDX2* **(D)** expression. Expression was normalized to *UBQ5* and data are the mean ± standard deviation of *n* = 3 biological replicates. Accessions are ordered according to leaf total vitamin B_6_ content as determined in Figure 5B. The effect of accession on gene expression was determined by one-way ANOVA (*α* = 0.05) with multiple comparisons and Tukey’s *post-hoc* test. Statistically significant differences between accessions are denoted by different letters. Correlation of *PDX1.3a* **(E)**, *PDX1.3b* **(F)**, *PDX1.3c* **(G)**, and *PDX2* **(H)** gene expression from panels **(A–D)**, respectively, with leaf total vitamin B_6_ contents in Figure 3B by Pearson’s correlation coefficient test (*p* < 0.05).

Previous studies observed correlations between the expression of vitamin biosynthesis genes and vitamin B_1_ contents in cassava (Mangel et al., 2017) and vitamin B_9_ in potatoes (Robinson et al., 2019). To understand the molecular determinants underlying contrasting vitamin B_1_ and B_6_ contents of the rice accessions characterized here, we chose to quantify transcript levels of selected genes for biosynthesis enzymes (see Figure 1 for vitamin B_1_ and B_6_ metabolism schemes). RNA was isolated from leaf tissues sampled for vitamin analyses and converted to cDNA for RT-qPCR assays of *THIC* for vitamin B_1,_ and *PDX1.3a*–*c* and *PDX2* for vitamin B_6_ (Figures 6, 7). A single copy of *THIC* is encoded in Nipponbare and IR64 genomes (Mangel et al., 2017; Qin et al., 2021), with *THIC* expression quantified in Kitaake and Tainung 67 accessions (Dong et al., 2016; Hsieh et al., 2021). Consistent with the model that vitamin B_1_ biosynthesis *de novo* in plants (Figure 1A) is predominantly localized to photosynthetic tissue (Guan et al., 2014; Colinas and Fitzpatrick, 2015), *THIC* expression was detected in leaves of all assayed rice accessions. *THIC* was differentially expressed across the accessions sampled [*F*(8, 18) = 18.94, *p* < 0.0001; Figure 6A]. To determine if tissue total vitamin B_1_ contents correlated with differential expression of *THIC* in leaves, total vitamin B_1_ content means (as determined by HPLC analyses in Figures 4B–D) were plotted against expression level means of *THIC* for each accession. Pearson’s correlation coefficient tests indicated total vitamin B_1_ contents in leaves did not correlate with expression at the RNA level of *THIC* (Figure 6B, R = 0.045, *R*^2^ = 0.002, *p* = 0.895). No correlation was observed between *THIC* expression in leaves and unpolished seed vitamin B_1_ contents (Figure 4C, R = 0.1614, *R*^2^ = 0.02605, *p* = 0.6783). *THIC* expression in leaves did, however, show a statistically significant, positive correlation with polished seed total vitamin B_1_ contents (Figure 6D, R = 0.7682, *R*^2^ = 0.5901, *p* = 0.0156). The *THIC* RT-qPCR primers used here bind in the coding region of *THIC* and do not discriminate between splice variants in the *THIC* 3′ UTR which contains a TDP riboswitch (Bocobza et al., 2007; Wachter et al., 2007; Noordally et al., 2020).

Vitamin B_6_ biosynthesis *de novo* in plants (Figure 1B) is considered to take place ubiquitously throughout plant tissues (Titiz et al., 2006). Three *PDX1.3* orthologs (*PDX1.3a*–*c*) are expressed in rice accessions Nipponbare and TP309 (Dell’Aglio et al., 2017; Mangel et al., 2019) and the encoded enzymes are predicted to be catalytically active (Moccand et al., 2014). A single *PDX2* locus is encoded in the Nipponbare and IR64 genomes and is expressed in TP309 (Mangel et al., 2019; Qin et al., 2021). Expression levels of these rice *PDX* genes were assayed by RT-qPCR using RNA samples isolated from leaf samples also used for HPLC quantification of vitamin B_6_ presented in Figure 5B. All four assayed *PDX* genes were expressed in leaves and are differentially expressed between the accessions *PDX1.3a*: [*F*(7, 16) = 17.29, *p* = 0.0301]; *PDX1.3b*: [*F*(7, 16) = 3.65, *p* = 0.0152]; *PDX1.3c*: [*F*(7, 16) = 3.946, *p* = 0.0109]; and *PDX2*: [*F*(7, 16) = 4.425, *p* = 0.0066; Figures 7A–D]. *PDX1.3b* was the most abundant *PDX1.3* transcript in leaves under these conditions, reaching levels over 50-fold higher than *PDX1.3c* for DNJ52 (Figures 7B,C). The mean of total vitamin B_6_ content was plotted against *PDX* gene expression for each accession for Pearson’s correlation coefficient tests (Figures 7E–H). *PDX1.3a–c* and *PDX2* expression levels showed no correlations with total vitamin B_6_ contents in leaves of the rice accessions sampled here (*p* > 0.05; Figures 7E–H). Unpolished and polished seed total vitamin B_6_ contents from Figures 5C,D were similarly plotted against *PDX* expression levels in leaves. No statistically significant correlations were observed between *PDX* expression levels in leaves and seed vitamin B_6_ contents (Supplementary Figure S2).

## Discussion

Previous studies reported natural variation for seed contents of total vitamin B_1_ across 121 accessions in rice germplasm (Villareal and Juliano, 1989; Sotelo et al., 1990; Kennedy and Burlingame, 2003), but such information is limited to three rice accessions for vitamin B_6_ contents in rice germplasm (Zarei et al., 2017). We profiled B_1_ and B_6_ vitamers using microbiological and HPLC assays in three tissue types (leaves, unpolished and polished grain) across a panel of 59 genetically diverse rice accessions, thereby expanding knowledge on these traits and providing insight into vitamer proportionality. The knowledge was complemented with an investigation of a select set of key genes involved in biosynthesis *de novo* of vitamin B_1_ and B_6_. This resource has the potential to inform on strategies for biofortification purposes as well as providing insight into possible regulatory pathways for vitamin content.

First for vitamin B_1_, seven accessions within our panel had been measured previously for unpolished and polished seed *total* vitamin B_1_ contents (Villareal and Juliano, 1989; Sotelo et al., 1990; Kennedy and Burlingame, 2003) and served as benchmarks in our screens (Figures 2, 4C,D; Supplemental Table 4). Similar values were obtained to those reported previously, despite employing different vitamin quantification techniques. Further, the relative distribution of the different B_1_ vitamers quantified here by HPLC across the three rice tissue types was consistent with previous studies (Dong et al., 2016; Strobbe et al., 2021a). Our study expands the list of accessions profiled for vitamin B_1_ contents and reinforces evidence that the genetic diversity of this trait in rice seeds is limited in the surveyed rice germplasm. Data collected so far indicate that immediate exploitation of rice germplasm is likely impractical for a breeding-based vitamin B_1_ biofortification strategy. The molecular basis underpinning vitamin B_1_ variation in staple crop germplasm has been investigated in cassava, wheat, and potato (Goyer and Haynes, 2011; Goyer and Sweek, 2011; Mangel et al., 2017; Li et al., 2018). In certain cassava accessions bearing duplications of *THI1* and *THIC* genes, leaf vitamin B_1_ contents are negatively correlated with transcript levels of Me*THIC2* (containing a functional TDP riboswitch) and Me*THI1b* (Mangel et al., 2017). Based on this finding, and similar observations for vitamin B_9_ in potato germplasm (Bali et al., 2018; Robinson et al., 2019), we sought to determine if this was also the case in rice as a potential molecular determinant underpinning vitamin B_1_ diversity (Figure 6). Clear differential expression of *THIC* transcripts in rice leaves was observed across the rice accessions contrasting in vitamin B_1_ contents (Figure 6A). Leaves are the principal site of vitamin B_1_ biosynthesis *de novo* (Fitzpatrick and Chapman, 2020). The expression pattern of *THIC* did not correlate with leaf total vitamin B_1_ contents under greenhouse conditions (Figure 6B). Yet, further correlation analyses with total vitamin B_1_ contents revealed a statistically significant, positive correlation between *THIC* expression in leaves and total vitamin B_1_ contents in polished seeds (Figure 6D). *THIC* encodes the chloroplast-localized HMP-P synthase and is the first enzyme committed to pyrimidine moiety biosynthesis *de novo* of vitamin B_1_ in plants (Figure 1A; Raschke et al., 2007; Coquille et al., 2013; Palmer and Downs, 2013)_._ *THIC* is functional in rice (Dong et al., 2016; Strobbe et al., 2021a). The promoter activity of Arabidopsis *THIC* is positively regulated by light (Raschke et al., 2007) and negatively regulated by the CIRCADIAN CLOCK ASSOCIATED 1 (CCA1) transcription factor that phases time of day circadian expression (Bocobza et al., 2013; Noordally et al., 2020). A TDP riboswitch is conserved in the 3′ UTR of *THIC* in higher plants and in Arabidopsis is subject to alternative mRNA 3′ processing in response to high or low nuclear TDP concentration (Raschke et al., 2007; Noordally et al., 2020), which produces stable or unstable transcripts through use of alternative polyadenylation sites (Bocobza et al., 2007; Wachter et al., 2007). The TDP riboswitch regulation of *THIC* is thought to be conserved in the green lineage (Bocobza et al., 2007; Croft et al., 2007; Wachter et al., 2007) and has been experimentally validated in both cassava *THIC* genes (Mangel et al., 2017). Arabidopsis *THIC* mRNA alternative 3′ processing is considered to fine-tune intracellular TDP supply in response to changes in demand for TDP-dependent enzymes throughout the day (Bocobza et al., 2013), independently of the circadian control of the *THIC* promoter (Bocobza et al., 2013; Noordally et al., 2020). A CCA1 binding site is present 28 bp upstream of the Nipponbare Os*THIC* 5′ UTR (Supplementary Figure S3), suggesting the Os*THIC* promoter could be subject to circadian regulation by CCA1. How high *THIC* expression in leaves of rice varieties might correspond to higher endosperm thiamine contents in the presence of a presumably functional TDP riboswitch in rice requires further investigation. With additional functional evidence, Os*THIC* relative expression levels in leaves might serve as a useful indicator of vitamin B_1_ contents in rice endosperm. TDP riboswitch activity in Os*THIC* needs to be confirmed by base editing, alongside analysis of *thiC* null mutant vitamer and developmental phenotypes. Based on Arabidopsis, rice, and maize models of vitamin B_1_ metabolism in plants, future work could characterize expression patterns of other genes encoding enzymes for vitamin B_1_ biosynthesis *de novo* in leaves (the main organ of biosynthesis *de novo* activity). For example, research efforts in vitamin B_1_ biofortification could explore the expression of the *TH1* gene responsible for condensation of the pyrimidine and thiazole heterocycles (Figure 1A) in rice endosperm, or candidate genes involved in transport or salvage pathways in tissues with minimal vitamin B_1_ biosynthesis *de novo* activity, such as seeds (Yazdani et al., 2013; Guan et al., 2014; Zallot et al., 2014; Dong et al., 2016). *THI1*, which encodes a single-turnover enzyme in thiazole moiety biosynthesis (Figure 1A), is functional in rice (Wang et al., 2006) and is sufficient to over-accumulate HET and vitamin B_1_ when ectopically expressed in transgenic rice (Dong et al., 2016; Strobbe et al., 2021a). *THI1* therefore represents an additional candidate gene and awaits characterization in diverse rice germplasm. Alternative sources of the thiazole moiety have been investigated (Sun et al., 2019) and could be exploited for biofortification purposes. It is clear nonetheless that an enhanced understanding of vitamin B_1_ metabolism is required in crops, including in rice, to permit successful endosperm biofortification at useful levels either by metabolic engineering or through identification of higher vitamin B_1_ content accessions other than those profiled here and previously (Villareal and Juliano, 1989; Sotelo et al., 1990; Kennedy and Burlingame, 2003; Dong et al., 2015; Hanson et al., 2018; Strobbe et al., 2021a). Given the high prevalence of suboptimal vitamin B_1_ status and deficiency disorders around the world, together with the wide consumption of vitamin B_1_-poor polished rice (Bhullar and Gruissem, 2013; Dhir et al., 2019; Johnson et al., 2019; Titcomb and Tanumihardjo, 2019; Whitfield et al., 2021), vitamin B_1_ biofortification of rice should be considered as a priority in assisting to combat micronutrient deficiency.

In contrast to vitamin B_1_, natural variation in vitamin B_6_ contents has not been extensively studied in rice germplasm with only three varieties quantified to date (Zarei et al., 2017), but no information is published on the molecular basis of such variation in rice. Vitamin B_6_ contents in germplasm of other crops have also received minimal attention, except for analyses in potatoes (Mooney et al., 2013; Goyer et al., 2019), wheat (Shewry et al., 2011; Freitag et al., 2018; Granda et al., 2018), and a small number of accessions of barley (Freitag et al., 2018; Granda et al., 2018), field beans (Freitag et al., 2018), and quinoa (Granda et al., 2018). Furthermore, regulation of vitamin B_6_ metabolism has not been widely investigated in monocot species compared to eudicots (Dell’Aglio et al., 2017; Yang et al., 2017; Mangel et al., 2019; Suzuki et al., 2020). Rice *PDX1* and *PDX2* genes are active (Dell’Aglio et al., 2017; Mangel et al., 2019) and are differentially expressed in transgenic lines over-expressing *MALATE DEHYDROGENASE 1*, concomitant with alterations to B_6_ vitamer profiles (Nan et al., 2020). Maize *PDX2* is functional (Yang et al., 2017; Suzuki et al., 2020) and is required for proper embryo development (Yang et al., 2017). Contrasting results from vitamin B_6_ biofortification efforts in model and crop plants that aimed at increasing vitamin B_6_ in target tissues through metabolic engineering indicates further research is needed to understand the regulation of vitamin B_6_ metabolism and sequestration, particularly in cereal endosperm (Chen and Xiong, 2009; Raschke et al., 2011; Li et al., 2015; Fudge et al., 2017; Mangel et al., 2019). Our results show that moderate natural variation does exist for vitamin B_6_ contents in rice germplasm (Figures 3, 5; Supplementary Tables S6, S9). Similar to vitamin B_1_, accessions profiled here with the highest vitamin B_6_ contents in polished seeds fall well below a practical level to justify introgression of such a trait for biofortification purposes (Figures 3, 5; Supplementary Table S9). Although no statistically significant correlations were observed between the expression in leaves of genes encoding for biosynthesis *de novo* enzymes and vitamin B_6_ contents under greenhouse conditions, future studies should expand such analyses to include analyses of vitamin B_6_ salvage pathway genes in leaves or expression analyses in the endosperm. Given the prevalence of vitamin B_6_ deficiency in certain populations, combined with the wide consumption of vitamin B_6_-poor polished rice, biofortification of rice with vitamin B_6_ also remains a priority in combatting micronutrient deficiency (Kim and Cho, 2014; Bird et al., 2017; Liu et al., 2019; Zhu et al., 2020).

A growing abundance of rice genetic resources such as the rice 3,000 genomes project (The 3000 rice genomes project, 2014; Wang et al., 2018), single-nucleotide polymorphism databases (McNally et al., 2009; Chebotarov et al., 2016), together with contemporary genome or base editing techniques (Zhu et al., 2017; Jin et al., 2019; Lu et al., 2020), remain alternative avenues to explore in order to biofortify rice endosperm with enhanced micronutrient contents. Redesigning the energetically costly vitamin B_1_ biosynthesis *de novo* pathway (Nelson et al., 2014) has also been proposed (Hanson et al., 2018; Sun et al., 2019) and could be drawn on for biofortification purposes.

In conclusion, our screen of diverse rice accessions under controlled greenhouse conditions advances our understanding of micronutrient trait diversity in rice germplasm by combining quantifications of total vitamin B_1_ and B_6_ levels as well as vitamer partition, over three tissue types of rice with insights into biosynthetic gene expression patterns. Further analyses using larger germplasm panels together with *in silico* genetic analyses such as genome-wide association studies (GWAS) might be useful for identifying accessions and loci to exploit for biofortification.

## Data Availability Statement

The raw data supporting the conclusions of this article will be made available by the authors, without undue reservation.

## Author Contributions

NM propagated and sampled the rice accessions, conducted yeast assays and RT-qPCR experiments, performed data analysis, and drafted figures and prepared tables. JBF performed the HPLC measurements and data analysis, prepared figures and tables, and wrote the manuscript. WG, TBF, and HV conceived the study, obtained funding, contributed to the analysis of the data, and edited manuscript drafts for submission. NM, JBF, WG, TBF, and HV commented and agreed on the final version of the manuscript and contributed to the article and approved the submitted version. The authors wish it to be known that NM and JBF are equal first authors and that WG, TBF, and HV are equal last and corresponding authors. For the purpose of their CVs, the respective authors can list their name as the first or as the last author. All authors contributed to the article and approved the submitted version.

## Funding

We gratefully acknowledge financial support from the Swiss National Science Foundation (grants 31003A-141117/1 and 31003A-162555 to TBF; grant 31003A-140911 to WG, HV, and TBF), the VELUX Foundation (WG), the Université de Genève (TBF), and ETH Zurich (WG). WG is supported by a Yushan Scholarship of the Ministry of Education in Taiwan.

## Conflict of Interest

The authors declare that the research was conducted in the absence of any commercial or financial relationships that could be construed as a potential conflict of interest.

## Publisher’s Note

All claims expressed in this article are solely those of the authors and do not necessarily represent those of their affiliated organizations, or those of the publisher, the editors and the reviewers. Any product that may be evaluated in this article, or claim that may be made by its manufacturer, is not guaranteed or endorsed by the publisher.

## Footnotes

^1^ https://www.genebanks.org/genebanks/irri/

^2^ www.mbkbase.org/rice/
