# Supplementary material for: Natural Variation in Vitamin B1 and Vitamin B6 Contents in Rice Germplasm
Source: Front Plant Sci. 2022 Apr 4;13:856880. doi: 10.3389/fpls.2022.856880 (PMC9014206; doi:10.3389/fpls.2022.856880)
Supplement: Supplementary file 1 [file Data_Sheet_1.docx]

Supplementary Material

**Supplementary Table 1. Description of the 59 accessions selected for quantification of vitamin B_1_ and B_6_ contents in experiment 1 and 2.**

Information about the selected accessions is reported in the International Rice Genebank Collection Information System (http://www.irgcis.irri.org:81/grc/SearchData.htm). The accessions TP309, IR64 and Nipponbare without IRGC accession number were obtained from the ETH Zurich (Swiss Federal Institute of Technology, Switzerland).

| **IRGC acc. number** | **Variety name** | **Source country** | **Varietal group** | **Rice blast** | **Bacterial blight** | **Sheath blight** | **Status of variety** | **Comments /**  **Related references** | |
| --- | --- | --- | --- | --- | --- | --- | --- | --- | --- |
| **9128** | **JW103** | India | Indica | Resistant | - | - | - | Wild species | |
| **9129** | **JW107** | India | Indica | Resistant | Moderately resistant | - | - | Wild species | |
| **29429** | **Tupa 147** | Bangladesh | Japonica | Resistant | Susceptible | Moderately susceptible | Breeding and inbred line | - | |
| **26139** | **No ordem lista 18 (IC)** | Brazil | Indica | Resistant | Resistant | - | - | - | |
| **26173** | **Saturn (Nova)** | Brazil | Japonica | Resistant | Susceptible | - | Released/Improved/  advanced cultivar | - | |
| **7655** | **Tjempo Welut** | Indonesia | Javanica | Resistant | Resistant | - | Breeding and inbred line | - | |
| **74** | **T 1** | India | Indica | Resistant | Susceptible | - | Released/Improved/  advanced cultivar | - | |
| **19672** | **CR133-47** | India | Japonica | Resistant | Susceptible | - | Breeding and inbred line | - | |
| **22327** | **ARC 12771** | India | Japonica | Resistant | Susceptible | - |  | - | |
| **530** | **Rei Shi Ko** | Japan | Japonica | Resistant | - | - |  | - | |
| **7344** | **Baek Huang Zo 59** | South Korea | Japonica | Resistant | - | - | Breeding and inbred line | - | |
| **32923** | **Ahmwe** | Myanmar | Indica | Resistant | Susceptible | - | - | - | |
| **606** | **Makapilay Lay Pusa B** | Philippines | Indica | Resistant | Susceptible | - | - | Villareal & Juliano (1989)  Kennedy & Burlingame (2003) | |
| **53065** | **Tapol** | Philippines | Indica | Resistant | Susceptible | Moderately susceptible | Landrace/  Traditional cultivar | Villareal & Juliano (1989)  Kennedy & Burlingame (2003) | |
| **5993** | **Carreon** | Philippines | Indica | Resistant | Susceptible | - | Breeding and inbred line | Villareal & Juliano (1989)  Kennedy & Burlingame (2003) | |
| **114** | **I-Kung-Pao** | Taiwan | Indica | Resistant | Susceptible | - | - | - | |
| **77** | **Hsinchu 56** | Taiwan | Japonica | Resistant | Susceptible | - | Breeding and inbred line | - | |
| **6893** | **Tainan-Iku 446** | Taiwan | Japonica | Resistant | Resistant | - | Breeding and inbred line | - | |
| **831** | **Gam Pai 30-12-15** | Taiwan | Indica | Resistant | Resistant | Moderately susceptible | Released/Improved/  advanced cultivar | - | |
| **-** | **IR64** | - | - | - | - | - | - | Villareal & Juliano (1989) | |
| **-** | **TP309** | - | - | - | - | - | - | - | |
| **8378** | **DNJ52** | Bangladesh | Indica | Resistant | Resistant | - | Breeding and inbred line | - | |
| **1190** | **Chin Ta 1-3-86** | China | Indica | Resistant | Susceptible | Moderately susceptible | Breeding and inbred line | - | |
| **2476** | **Araji** | Japan | Indica | Resistant | Susceptible | - | - | - | |
| **33130** | **Indane** | Myanmar | Japonica | Resistant | Susceptible | - | Breeding and inbred line | - | |
| **16150** | **Phulpata** | Nepal | Indica | Resistant | Susceptible | Moderately resistant | - | - |  |
| **16170** | **Aanga** | Nepal | Indica | Resistant | - | Susceptible | - | - |  |
| **172** | **Nanhng Mon S 4** | Thailand | Indica | Resistant | Susceptible | Moderately susceptible | Breeding and inbred line | - |  |
| **10585** | **Lua Chien**  **(C 6583)** | Vietnam | Indica | Resistant | - | Moderately susceptible | Breeding and inbred line | - |  |
| **-** | **Nipponbare** | - | - | - | - | - | - | - |  |
| **1029** | **Fan Ho Ku** | China | Indica | Resistant | - | - | Landrace/  Traditional cultivar | - |  |
| **1049** | **Chang Li** | China | Indica | Resistant | - | - | Landrace/  Traditional cultivar | - |  |
| **2283** | **Wase Sekitoro C** | South Korea | Japonica | Resistant | Susceptible | Moderately susceptible | Landrace/  Traditional cultivar |  |  |
| **19751** | **Han Yang Zo** | South Korea | Japonica | Resistant | Susceptible | - | Landrace/  Traditional cultivar | - |  |
| **11442** | **Kamod** | Nepal | Indica | Resistant | - | - | Landrace/  Traditional cultivar | - |  |
| **23787** | **Anandi** | Nepal | Javanica | Resistant | Susceptible | - | Landrace/  Traditional cultivar | - |  |
| **14981** | **Magawk-Pi 269-7-22** | Thailand | Javanica | Resistant | Susceptible | Moderately susceptible | Landrace/  Traditional cultivar | - |  |
| **15031** | **Daw Magawk** | Thailand | Indica | Resistant | Susceptible | Moderately susceptible | Landrace/  Traditional cultivar | - |  |
| **6997** | **Cau Phu Xuyen 264** | Vietnam | Indica | Resistant | Moderately resistant | - | Landrace/  Traditional cultivar | - |  |
| **4183** | **Mapili** | Indonesia | Indica | Resistant | Susceptible | - | Landrace/  Traditional cultivar | - |  |
| **13524** | **Hawara Batu** | Indonesia | Javanica | Resistant |  | - | Landrace/  Traditional cultivar | - |  |
| **2764** | **E-Kha-Keha** | Thailand | Japonica | Resistant | Susceptible | - | Landrace/  Traditional cultivar | - |  |
| **2802** | **Payaipatosu 4** | Thailand | Japonica | Resistant |  | Moderately susceptible | Landrace/  Traditional cultivar | - |  |
| **9864** | **Shaeta** | Bangladesh | Indica | Resistant | Susceptible |  | Landrace/  Traditional cultivar | - |  |
| **25860** | **Hanumanjata** | Bangladesh | Indica | Resistant | Resistant |  | Landrace/  Traditional cultivar | - |  |
| **4010** | **Tung-Huan-Pe-18** | Philippines | Indica | Resistant | - | Moderately susceptible | Landrace/  Traditional cultivar | - |  |
| **3663** | **Bansi** | India | Indica | Resistant | - | Moderately susceptible | Landrace/  Traditional cultivar | - |  |
| **9933** | **Vaid Butti** | India | Indica | Resistant | - | Moderately susceptible | Landrace/  Traditional cultivar | - |  |
| **51107** | **Juchitan A74** | Mexico | Hybrid | - | Susceptible | Moderately susceptible | - | Sotelo *et al* (1990)  Kennedy & Burlingame (2003) |  |
| **117281** | **Aswina** | Bangladesh | - | - | - | - | - | McNally *et al* (2009) |  |
| **117264** | **Azucena** | Philippines | - | - | - | - | - | Villareal & Juliano (1989)  Kennedy & Burlingame (2003)  McNally *et al.* (2009) |  |
| **117266** | **Dular** | India | - | - | - | - | - | McNally *et al.* (2009) |  |
| **117267** | **FR 13 A** | India | - | - | - | - | - | McNally *et al.* (2009) |  |
| **117268** | **IR 64-21** | Philippines | - | - | - | - | - | McNally *et al.* (2009) |  |
| **117274** | **Nipponbare** | Japan | - | - | - | - | - | McNally *et al.* (2009) |  |
| **117275** | **Pokkali** | India | - | - | - | - | - | McNally *et al.* (2009) |  |
| **117276** | **Sadu Cho** | South Korea | - | - | - | - | - | McNally *et al.* (2009) |  |
| **117279** | **Tainung 67** | Taiwan | - | - | - | - | - | McNally *et al.* (2009) |  |
| **117280** | **Zhenshan 97 B** | China | - | - | - | - | - | McNally *et al.* (2009) |  |

## Supplementary Table 2. List of oligonucleotide primers used in this study for qRT-PCR. MSU RGAP release 7 locus identifiers are listed below gene names.

| **Locus** | **Sequence of oligonucleotide (5' to 3')** | **Reference** |
| --- | --- | --- |
| Os*UBQ5* | ACCACTTCGACCGCCACTACT | Jain *et al* (2006) |
| Os01g22490 | ACGCCTAAGCCTGCTGGTT |  |
| Os*THI1*  Os07g34570 | TTCCACTGCCACCGTGTGT  GAGACGGATCCATGGAAGTTTTC | This study |
|  |  |  |
| Os*THIC*  Os03g47610 | TTTGACACATACGACACCAG  GACCACCTAACTTCTCCCTC | This study |
|  |  |  |
| Os*PDX1.3a*  Os07g01020 | GCCTTATCCTTCTTTCGCTA  CTGGCTGGCTGGTGTCTAATTC | Dell'Aglio *et al* (2017) |
|  |  |  |
| Os*PDX1.3b* | TTGGCATCAACCTCTCCG  AGGAAGGATGGCACTCTACG | Dell'Aglio *et al.* (2017) |
| Os10g01080 |  |  |
| Os*PDX1.3c*  Os11g48080 | GTCGGCATCAACCTCAACG  AGAGGGAGCAGGAGCAGGA | Dell'Aglio *et al.* (2017) |
| Os*PDX2*  Os02g03740 | CCCATCTTCGAGTAGCTCATG  CTCAACTTTCTCTATTGTCACC | Dell'Aglio *et al.* (2017) |

**Supplementary Table 3. Threshold cycle (C_t_) values for UBQ5, the reference gene used for qRT-PCR, across sampled accessions.**

| **For *THIC*** | |  | **For *PDX1, PDX2*** | |
| --- | --- | --- | --- | --- |
| **Sample name** | ***UBQ5* Ct value** |  | **Sample name** | ***UBQ5* Ct value** |
| I-Kung-Pao-1 | 24.67 |  | DNJ52-1 | 24.35 |
|  | 24.37 |  |  | 24.32 |
| I-Kung-Pao-2 | 24.44 |  | DNJ52-2 | 24.32 |
|  | 24.59 |  |  | 24.55 |
| I-Kung-Pao-3 | 24.61 |  | DNJ52-3 | 24.03 |
|  | 24.67 |  |  | 24.12 |
| IR64-1 | 24.74 |  | I-Kung-Pao-1 | 24.51 |
|  | 24.71 |  |  | 24.54 |
| IR64-2 | 24 |  | I-Kung-Pao-2 | 24.46 |
|  | 24.09 |  |  | 24.33 |
| IR64-3 | 24.22 |  | I-Kung-Pao-3 | 24.61 |
|  | 24.34 |  |  | 24.61 |
| Daw Magawk-1 | 23.59 |  | IR64-1 | 24.66 |
|  | 23.65 |  |  | 24.73 |
| Daw Magawk-2 | 23.64 |  | IR64-2 | 24.01 |
|  | 23.57 |  |  | 23.99 |
| Daw Magawk-3 | 23.36 |  | IR64-3 | 24.24 |
|  | 23.6 |  |  | 24.44 |
| Aanga-1 | 24.81 |  | Daw Magwak-1 | 23.73 |
|  | 24.71 |  |  | 23.63 |
| Aanga-2 | 24.32 |  | Daw Magwak-2 | 23.58 |
|  | 24.31 |  |  | 23.59 |
| Aanga-3 | 24.81 |  | Daw Magwak-3 | 23.46 |
|  | 24.94 |  |  | 23.44 |
| Hanumanjata-1 | 23.71 |  | Tapol-1 | 24.35 |
|  | 23.74 |  |  | 24.31 |
| Hanumanjata-2 | 23.85 |  | Tapol-2 | 24.07 |
|  | 23.79 |  |  | 24.06 |
| Hanumanjata-3 | 23.92 |  | Tapol-3 | 24.77 |
|  | 23.98 |  |  | 24.79 |
| Shaeta-1 | 24 |  | Aanga-1 | 24.7 |
|  | 24.01 |  |  | 24.7 |
| Shaeta-2 | 24.33 |  | Aanga-2 | 24.16 |
|  | 24.3 |  |  | 24.23 |
| Shaeta-3 | 24.55 |  | Aanga-3 | 24.67 |
|  | 24.5 |  |  | 24.78 |
| Vaid Butti-1 | 23.11 |  | Nipponbare-1 | 23.69 |
|  | 23.2 |  |  | 23.72 |
| Vaid Butti-2 | 23.02 |  | Nipponbare-2 | 23.57 |
|  | 23.04 |  |  | 23.68 |
| Vaid Butti-3 | 23.35 |  | Nipponbare-3 | 23.44 |
|  | 23.29 |  |  | 23.52 |
| Fan Ho Ku-1 | 24.14 |  | TP309-1 | 23.58 |
|  | 24.02 |  |  | 23.58 |
| Fan Ho Ku-2 | 24.34 |  | TP309-2 | 23.86 |
|  | 24.23 |  |  | 23.87 |
| Fan Ho Ku-3 | 23.99 |  | TP309-3 | 23.88 |
|  | 23.91 |  |  | 23.9 |
| Juchitan-1 | 24.18 |  |  |  |
|  | 24.51 |  | **Mean** | 24.13 |
| Juchitan-2 | 23.61 |  | **Standard deviation** | 0.43 |
|  | 23.74 |  |  |  |
| Juchitan-3 | 24.54 |  |  |  |
|  | 24.46 |  |  |  |
|  |  |  |  |  |
| **Mean** | 24.08 |  |  |  |
| **Standard deviation** | 0.50 |  |  |  |

**Supplementary Table 4. Phenotypic characterization of rice accessions grown under greenhouse conditions.**

Experiment 1: Evaluation of plant phenotype at maturity of 49 selected rice accessions. Mean ± SD of 3 biological replicates, except for Nipponbare (n = 6), IR64 (n = 6), TP309 (n = 6). Tukey’s multiple comparison test (*p* < 0.05).

Experiment 2: Evaluation of plant phenotype at maturity of 21 rice accessions selected amongst the 49 tested in experiment 1 and of 10 additional accessions. Mean ± SD of 3 biological replicates, except for Nipponbare (n = 6), IR64 (n = 6), TP309 (n = 6), (OSNP) IR 64-21 (n = 2), (OSNP) Nipponbare (n = 2). Tukey’s multiple comparison test (*p* < 0.05).

|  | | **Experiment 1** | | | | | | **Experiment 2** | | | | |  |  |
| --- | --- | --- | --- | --- | --- | --- | --- | --- | --- | --- | --- | --- | --- | --- |
|  |  | **Plant height**  **(cm)** | **Number of panicles**  **/plant** | | **Leaf dry weight (g)** | | | **Plant height (cm)** | **Leaf dry weight (g)** | | **Number of panicles**  **/plant** | |  |  |
| **JW103** | | | | - | **7.3**^bcdefg^  [±0.6] | | **19.6**^defgh^  [±1.4] | | |  |  | |  | |
| **JW107** | | | | **149.3**^hijklm^  [±5.0] | **7.0**^abcdef^  [±2.0] | | **18.4**^bcdefgh^  [±5.7] | | | **118.7**^cd^  [±4.9] | **17.6**^bcdefgh^  [±3.1] | | **9.0**^efg^  [±1.0] | |
| **Tupa 147** | | | | **169.3**^jklmno^  [±5.5] | **12.3**^g^  [±3.5] | | **24.2**^ghi^  [±3.7] | | |  |  | |  | |
| **No ordem lista 18(IC)** | | | | **112.3**^abcde^  [±4.7] | **4.3**^abcde^  [±0.6] | | **11.9**^abcdef^  [±2.7] | | |  |  | |  | |
| **Saturn (Nova)** | | | | **128.0**^cdefgh^  [±1.0] | **3.7**^abcd^  [±0.6] | | **10.5**^abcde^  [±1.1] | | |  |  | |  | |
| **Tjempo Welut** | | | | **139.7**^efghi^  [±0.6] | **3.0**^abc^  [±0.0] | | **16.9**^abcdefgh^  [±0.8] | | |  |  | |  | |
| **T 1** | | | | **141.7**^fghij^  [±2.9] | **7.7**^cdefg^  [±4.0] | | **24.2**^fghi^  [±5.5] | | |  |  | |  | |
| **CR133-47** | | | | **119.3**^bcdefg^  [±3.8] | **7.0**^bcdef^  [±1.0] | | **9.6**^abcde^  [±1.5] | | |  |  | |  | |
| **ARC 12771** | | | | **-** | **4.7**^abcde^  [±0.6] | | **14.9**^abcdefgh^  [±2.2] | | |  |  | |  | |
| **Rei Shi Ko** | | | | **114.7**^abcdef^  [±5.5] | **8.3**^defg^  [±2.3] | | **11.7**^abcdef^  [±1.5] | | | **118.7**^cd^  [±3.8] | **14.5**^abcdefg^  [±1.2] | | **10.7**^g^  [±1.5] | |
| **Baek Huang Zo 59** | | | | **181.0**^nop^  [±15.9] | **4.7**^abcde^  [±1.2] | | **13.2**^abcdefg^  [±0.4] | | |  |  | |  | |
| **Ahmwe** | | | | **163.7**^ijklmno^  [±15.0] | **10.3**^fg^  [±0.6] | | **20.8**^efgh^  [±8.0] | | |  |  | |  | |
| **Makapilay Pusa B** | | | | **155.7**^hijklmn^  [±5.1] | **6.0**^abcdef^  [±1.0] | | **19.6**^cdefgh^  [±3.8] | | | **155.0**^hijk^  [±1.7] | **28.8**^hij^  [±2.0] | | **5.7**^abcdef^  [±0.6] | |
| **Tapol** | | | | **186.7**^op^  [±10.4] | **4.3**^abcde^  [±1.5] | | **25.1**^ghi^  [±2.9] | | | **153.0**^ghijk^  [±2.6] | **23.9**^ghi^  [±5.6] | | **8.3**^defg^  [±1.5] | |
| **Carreon** | | | | **155.3**^hijklmn^  [±8.5] | **5.3**^abcdef^  [±3.2] | | **16.0**^abcdefgh^  [±7.8] | | | **123.3**^de^  [±3.2] | **16.4**^bcdefg^  [±3.0] | | **6.0**^abcdef^  [±2.0] | |
| **I-Kung-Pao** | | | | **130.3**^cdefgh^  [±8.0] | **5.3**^abcdef^  [±1.2] | | **19.7**^defgh^  [±4.8] | | | **126.0**^de^  [±1.0] | **13.3**^abcdefg^  [±2.1] | | **6.7**^bcdefg^  [±1.2] | |
| **Hsinchu 56** | | | | **107.7**^abcd^  [±3.1] | **4.3**^abcde^  [±0.6] | | **10.0**^abcde^  [±1.5] | | |  |  | |  | |
| **Tainan-Iku 446** | | | | **112.0**^abcde^  [±4.4] | **4.7**^abcde^  [±1.2] | | **12.8**^abcdefg^  [±1.2] | | |  |  | |  | |
|  | | **Plant height (cm)** | | | **Number of panicles/plant** | | **Leaf dry weight (g)** | **Plant height (cm)** | | | **Leaf dry weight (g)** | | **Number of panicles/ plant** |  |
| **Gam Pai 30-12-15** | | | **174.7**^mnop^  [±18.2] | | | **5.3**^abcdef^  [±1.5] | | **40.1**^j^  [±5.9] |  | | |  | |  |
| **DNJ52** | | | **136.7**^efghi^  [±2.1] | | | **4.7**^abcde^  [±1.5] | | **15.1**^abcdefgh^  [±2.6] | **160.7**^jkl^  [±6.0] | | | **12.4**^abcdefg^  [±2.8] | | **10.4**^bcde^  [±2.0] |
| **Chin Ta 1-3-86** | | | **138.3**^efghi^  [±10.4] | | | **12.3**^g^  [±5.5] | | **14.5**^abcdefgh^  [±4.2] | **145.7**^efghij^  [±4.0] | | | **17.8**^cdefgh^  [±0.9] | | **14.1**^e^  [±0.4] |
| **Araji** | | | **176.7**^mnop^  [±7.8] | | | **7.0**^bcdef^  [±1.0] | | **24.4**^ghi^  [±6.4] |  | | |  | |  |
| **Indane** | | | **152.3**^hijklmn^  [±4.0] | | | **3.3**^abcd^  [±0.6] | | **16.6**^abcdegh^  [±2.3] |  | | |  | |  |
| **Phulpata** | | | **175.7**^mnop^  [±7.5] | | | **5.7**^abcdef^  [±0.6] | | **23.6**^fghi^  [±1.4] | **151.3**^fghij^  [±8.6] | | | **22.5**^fghi^  [±3.1] | | **0.5**^a^  [±0.3] |
| **Aanga** | | | **172.0**^klmnop^  [±2.0] | | | **7.0**^bcdef^  [±1.0] | | **17.9**^bcdefgh^  [±1.6] | **156.0**^ijkl^  [±1.7] | | | **13.7**^abcdefg^  [±1.6] | | **8.0**^abcde^  [±2.0] |
| **Nanhng Mon S 4** | | | **172.7**^lmnop^  [±11.4] | | | **4.0**^abcde^  [±1.0] | | **24.2**^ghi^  [±4.0] |  | | |  | |  |
| **Lua Chien (C 6583)** | | | **143.7**^ghijk^  [±3.5] | | | **9.0**^efg^  [±1.0] | | **17.6**^bcdefgh^  [±1.7] |  | | |  | |  |
| **Fan Ho Ku** | | | **131.7**^cdefgh^  [±13.3] | | | **4.7**^abcde^  [±1.2] | | **8.5**^abcd^  [±2.8] | **138.7**^defghi^  [±2.1] | | | **15.2**^abcdefg^  [±2.4] | | **13.9**^e^  [±2.3] |
| **Chang Li** | | | **150.3**^hijklm^  [±10.3] | | | **4.0**^abcde^  [±0.0] | | **7.1**^ab^  [±1.5] |  | | |  | |  |
| **Wase Sekitoro C** | | | **161.7**^ijklmno^  [±7.2] | | | **3.7**^abcd^  [±0.6] | | **8.7**^abcd^  [±4.4] |  | | |  | |  |
| **Han Yang Zo** | | | **129.0**^cdefgh^ [±8.2] | | | **4.0**^abcde^  [±1.0] | | **15.1**^abcdefgh^  [±7.0] | **126.7**^de^  [±13.1] | | | **12.8**^abcdefg^  [±6.9] | | **11.3**^bcde^  [±6.2] |
| **Kamod** | | | **154.0**^hijklmn^  [±5.6] | | | **4.3**^abcde^  [±0.6] | | **9.5**^abcde^  [±1.1] |  | | |  | |  |
| **Anandi** | | | **129.0**^cdefgh^  [±7.2] | | | **3.0**^abc^  [±0.0] | | **7.2**^ab^  [±1.0] |  | | |  | |  |
| **Magawk-Pi 269-7-22** | | | **133.0**^defghi^  [±6.1] | | | **3.0**^abc^  [±0.0] | | **13.3**^abcdefg^  [±0.7] |  | | |  | |  |
| **Daw Magawk** | | | **170.7**^klmno^  [±26.0] | | | **1.7**^a^  [±1.5] | | **17.0**^abcdefgh^  [±5.6] | **135.7**^defgh^  [±4.0] | | | **15.9**^bcdefg^  [±0.5] | | **3.5**^abc^  [±0.5] |
| **Cau Phu Xuyen 264** | | | **154.7**^hijklmn^  [±4.0] | | | **5.0**^abcde^  [±1.0] | | **14.1**^abcdefgh^  [±2.9] |  | | |  | |  |
| **Mapili** | | | **156.7**^hijklmn^  [±9.9] | | | **4.3**^abcde^  [±1.2] | | **20.7**^efgh^  [±2.2] | **133.3**^defg^  [±6.4] | | | **21.3**^efghi^  [±2.0] | | **7.5**^abcde^  [±1.1] |
| **Hawara Batu** | | | **210.0**^q^  [±0.0] | | | **2.7**^ab^  [±0.6] | | **31.3**^ij^  [±5.0] |  | | |  | |  |
| **E-Kha-Keha** | | | **144.7**^ghijkl^  [±14.0] | | | **4.3**^abcde^  [±1.5] | | **8.1**^abc^  [±1.9] |  | | |  | |  |

|  | **Plant height (cm)** | **Number of panicles/ plant** | **Leaf dry weight (g)** | **Plant height (cm)** | **Leaf dry weight (g)** | **Number of panicles/**  **plant** |  |
| --- | --- | --- | --- | --- | --- | --- | --- |
| **Payaipatosu 4** | | **136.3**^defghi^  [±9.9] | **3.7**^abcd^  [±2.1] | **7.6**^ab^  [±2.6] |  |  |  |
| **Shaeta** | | **143.3**^fghijk^  [±1.5] | **4.0**^abcde^  [±0.0] | **5.6**^a^  [±0.3] | **130.7**^de^  [±4.7] | **15.1**^abcdefg^  [±0.6] | **9.7**^efgh^  [±0.6] |
| **Hanumanjata** | | **143.7**^ghijk^  [±3.1] | **3.7**^abcd^  [±0.6] | **7.6**^ab^  [±1.3] | **131.7**^def^  [±6.0] | **15.2**^abcdefg^  [±1.2] | **9.7**^efgh^  [±0.6] |
| **Tung-Huan-Pe 18** | | **140.7**^efghij^  [±4.0] | **4.0**^abcde^  [±1.0] | **8.4**^abcd^  [±1.5] |  |  |  |
| **Bansi** | | **196.0**^pq^  [±15.1] | **3.3**^abcd^  [±0.6] | **13.4**^abcdefg^  [±0.9] |  |  |  |
| **Vaid Butti** | | **141.7**^fghij^ [±9.0] | **6.3**^abcdef^  [±1.5] | **16.5**^bcdefgh^  [±5.2] | **137.3**^defghi^  [±2.5] | **22.8**^fghi^  [±4.1] | **9.3**^efgh^  [±0.6] |
| **Juchitan A74** | | **95.7**^ab^  [±1.2] | **4.3**^abcde^  [±0.6] | **15.8**^abcdefgh^  [±1.7] | **78.3**^a^  [±1.2] | **11.1**^abcdef^  [±1.3] | **5.0**^abcd^  [±0.0] |
| **Nipponbare** | | **97.5**^ab^  [±8.7] | **4.7**^abcde^  [±1.0] | **9.9**^abc^  [±4.0] | **75.2**^ab^  [±13.9] | **5.3**^abcdef^  [±3.0] | **5.3**^bcdefgh^  [±1.6] |
| **TP309** | | **107.5**^abc^  [±4.3] | **4.3**^abcd^  [±0.5] | **11.4**^abcde^  [±1.4] | **105.7**^bc^  [±4.1] | **11.5**^abcde^  [±5.7] | **5.3**^abcd^  [±2.7] |
| **IR64** | | **93.8**^a^  [±5.0] | **7.2**^bcde^  [±1.3] | **11.3**^abcde^  [±1.8] | **86.5**^a^  [±3.8] | **6.4**^ab^  [±1.4] | **6.5**^abcdef^  [±1.8] |
| **(O. SNP) Aswina** | |  |  |  | **176.0**^lm^  [±9.0] | **32.8**^hij^  [±4.5] | **7.0**^abcdefg^  [±1.0] |
| **(O. SNP) Azucena** | |  |  |  | **172.7**^klm^  [±8.4] | **16.6**^bcdefg^  [±3.2] | **4.0**^ab^  [±0.0] |
| **(O. SNP) Dular** | |  |  |  | **134.3**^defg^  [±3.8] | **15.4**^abcdefg^  [±2.4] | **8.3**^cdefgh^  [±0.6] |
| **(O. SNP) FR 13 A** | |  |  |  | **119.0**^cd^  [±3.6] | **37.2**^hij^  [±12.1] | **12.0**^h^  [±2.0] |
| **(O. SNP) Nipponbare** | |  |  |  | **87.0**^a^  [±1.4] | **10.0**^a^  [±1.2] | **8.5**^bcdefgh^  [±0.7] |
| **(O. SNP) Pokkali** | |  |  |  | **187.7**^lm^  [±1.5] | **32.9**^hij^  [±2.5] | **5.7**^abcde^  [±1.2] |
| **(O. SNP) Sadu Cho** | |  |  |  | **131.3**^def^  [±3.2] | **19.3**^defgh^  [±1.5] | **10.0**^fgh^  [±1.0] |
| **(O. SNP) Tainung 67** | |  |  |  | **87.7**^a^  [±9.6] | **8.8**^abcd^  [±1.4] | **4.3**^abcd^  [±0.6] |
| **(O. SNP) Zhenshan 97 B** | |  |  |  | **96.7**^ab^  [±2.5] | **6.8**^abc^  [±2.1] | **5.7**^abcde^  [±0.6] |
| **(O. SNP) IR 64-21** | |  |  |  | **82.5**^ab^  [±9.2] | **7.3**^abcd^  [±3.9] | **4.5**^abcd^  [±2.1] |

**Supplementary Table 5.** Experiment 1 vitamin B_1_ contents in leaves **(A)**, unpolished seeds **(B)** and polished seeds **(C)** in rice accessions grown under greenhouse conditions and quantified by a microbiological (yeast) assay. The accessions with vitamin B_1_ content below the 25^th^ percentile of the distribution were considered as low vitamin B_1_ accessions and those with vitamin content above the 75^th^ percentile were considered as high vitamin B_1_ accessions. Low, intermediate and high vitamin B_1_accessions selected for experiment 2 are in bold. Mean ± SD of 3 biological replicates, except Nipponbare (n = 6), IR64 (n = 6) and TP309 (n = 6) for the three tissues; No ordem lista 18 (IC) (n = 2) and Bansi (n = 2) for leaves; Daw Magawk (n = 2) for unpolished seeds, Daw Magawk (n = 2) and Cau Phu Xuyen 264 (n = 2) for polished seeds. Tukey's multiple comparison test (*p* < 0.05).

| **A Leaves** | | |  | **B Unpolished seeds** | | |  | **C Polished seeds** | | |
| --- | --- | --- | --- | --- | --- | --- | --- | --- | --- | --- |
| **Accession** | **Vitamin B_1_ (ng mg FW^-1^)** | |  | **Accession** | **Vitamin B_1_ (ng mg DW^-1^)** | |  | **Accession** | **Vitamin B_1_ (ng mg DW^-1^)** | |
| **Hanumanjata** | **1.11** [±0.23]^a^ | Below the 25^th^ percentile of vitamin B_1_ content distribution |  | **Vaid Butti** | **2.35** [±0.08]^a^ | Below the 25^th^ percentile of vitamin B_1_ content distribution |  | Hsinchu 56 | **0.65** [±0.04]^a^ | Below the 25^th^ percentile  of vitamin B_1_ content distribution |
| Bansi | **1.28** [±0.04]^abcd^ |  |  | **Mapili** | **3.23** [±0.28]^ab^ |  |  | **I-Kung-Pao** | **0.65** [±0.07]^a^ |  |
| **Shaeta** | **1.32** [±0.43]^ab^ |  |  | **DNJ52** | **3.95** [±0.44]^abc^ |  |  | Tainan-Iku 446 | **0.68** [±0.10]^ab^ |  |
| **JW107** | **1.42** [±0.08]^abc^ |  |  | **Tapol** | **4.04** [±0.91]^abcd^ |  |  | Gam Pai 30-12-15 | **0.69** [±0.02]^ab^ |  |
| Magwak-Pi 269-7-22 | **1.44** [±0.12]^abc^ |  |  | T 1 | **4.37** [±0.94]^abcde^ |  |  | Wase Sekitoro C | **0.72** [±0.08]^abc^ |  |
| **Han Yang Zo** | **1.46** [±0.24]^abcd^ |  |  | Tung-Huan-Pe 18 | **4.44** [±0.16]^abcdef^ |  |  | Hawara Batu | **0.72** [±0.07]^abcd^ |  |
| Chang Li | **1.56** [±0.35]^abcde^ |  |  | Hawara Batu | **4.56** [±0.29]^abcdefg^ |  |  | Anandi | **0.73** [±0.03]^abcd^ |  |
| Payaipatosu 4 | **1.61** [±0.48]^abcdef^ |  |  | Bansi | **4.72**[±0.43]^abcdefgh^ |  |  | **Vaid Butti** | **0.74** [±0.05]^abcde^ |  |
| Anandi | **1.70** [±0.44]^abcdef^ |  |  | **JW107** | **4.79** [±0.33]^bcdefgh^ |  |  | Ahmwe | **0.75** [±0.08]^abcde^ |  |
| Hawara Batu | **1.72** [±0.27]^abcdef^ |  |  | **Nipponbare** | **4.84** [±1.53]^bcdefg^ |  |  | **Makapilay Pusa B** | **0.75** [±0.14]^abcde^ |  |
| Wase Sekitoro C | **1.82** [±0.39]^abcdefg^ |  |  | **Hanumanjata** | **4.88** [±0.48]^bcdefgh^ |  |  | **Fan Ho Ku** | **0.75** [±0.06]^abcdef^ |  |
| Kamod | **1.86** [±0.69]^abcdefg^ | Between 25^th^ and 75^th^ percentile of  vitamin B_1_ content distribution |  | **IR64** | **4.91** [±0.59]^bcdefg^ |  |  | **Chin Ta 1-3-86** | **0.75** [±0.09]^abcdef^ |  |
| **DNJ52** | **1.86** [±0.44]^abcdefg^ |  |  | **TP309** | **4.94** [±0.96]^bcdefgh^ | Between 25^th^ and 75^th^ percentile of  vitamin B_1_ content distribution |  | **IR64** | **0.76** [±0.09]^abc^ | Between 25^th^ and 75^th^ percentile of  vitamin B_1_ content distribution |
| Nanhng Mon S 4 | **1.89** [±0.29]^abcdefg^ |  |  | **Makapilay Pusa B** | **5.06** [±0.31]^bcdefghi^ |  |  | **DNJ52** | **0.77** [±0.12]^abcdefg^ |  |
| **Daw Magawk** | **1.90** [±0.15]^abcdefg^ |  |  | Magwak-Pi 269-7-22 | **5.12** [±0.33]^bcdefghi^ |  |  | Tung-Huan-Pe 18 | **0.78** [±0.11]^abcdefg^ |  |
| **Fan Ho Ku** | **1.92** [±0.46]^abcdefg^ |  |  | **Chin Ta 1-3-86** | **5.13** [±0.70]^bcdefghi^ |  |  | Magwak-Pi 269-7-22 | **0.79** [±0.14]^abcdefg^ |  |
| JW103 | **1.93** [±0.54]^abcdefg^ |  |  | JW103 | **5.17** [±0.61]^bcdefghi^ |  |  | E-Kha-Keha | **0.80** [±0.07]^abcdefgh^ |  |
| E-Kha-Keha | **1.98** [±0.63]^abcdefg^ |  |  | Nanhng Mon S 4 | **5.20** [±0.52]^bcdefghi^ |  |  | Chang Li | **0.81** [±0.08]^abcdefgh^ |  |
| No ordem lista 18 (IC) | **2.02** [±0.35]^abcdefghi^ |  |  | **Han Yang Zo** | **5.26** [±0.64]^bcdefghi^ |  |  | Araji | **0.82** [±0.03]^abcdefgh^ |  |
| Cau Phu Xuyen 264 | **2.07** [±0.58]^abcdefgh^ |  |  | Gam Pai 30-12-15 | **5.27** [±0.35]^bcdefghi^ |  |  | **TP309** | **0.82** [±0.17]^abcdefg^ |  |
| **Aanga** | **2.08** [±0.44]^abcdefgh^ |  |  | NO ORDEM LISTA 18(IC) | **5.29** [±0.53]^bcdefghi^ |  |  | **Nipponbare** | **0.85** [±0.12]^abcdefg^ |  |
| Tainan-Iku 446 | **2.17** [±0.25]^abcdefghi^ |  |  | **I-Kung-Pao** | **5.37** [±0.34]^bcdefghi^ |  |  | Cau Phu Xuyen 264 | **0.85** [±0.04]^abcdefghi^ |  |
| Araji | **2.18** [±0.32]^abcdefghi^ |  |  | **Shaeta** | **5.43** [±0.45]^bcdefghi^ |  |  | Bansi | **0.87** [±0.03]^abcdefghi^ |  |
| Gam Pai 30-12-15 | **2.23** [±0.58]^abcdefghi^ |  |  | Hsinchu 56 | **5.51** [±0.16]^bcdefghi^ |  |  | Payaipatosu 4 | **0.90** [±0.03]^abcdefghi^ |  |
| **Vaid Butti** | **2.28** [±0.77]^abcdefghi^ |  |  | **Carreon** | **5.52** [±0.17]^bcdefghi^ |  |  | LUA CHIEN (C 6583) | **0.92** [±0.07]^abcdefghi^ |  |
| Tjempo Welut | **2.29** [±0.21]^abcdefghi^ |  |  | CR133-47 | **5.60** [±0.28]^bcdefghij^ |  |  | **Daw Magawk** | **0.96** [±0.12]^abcdefghij^ |  |
| **Nipponbare** | **2.33** [±0.40]^abcdefgh^ |  |  | LUA CHIEN (C 6583) | **5.72** [±0.52]^cdefghij^ |  |  | **Mapili** | **0.99** [±0.12]^abcdefghij^ |  |
| Tung-Huan-Pe 18 | **2.41** [±1.30]^abcdefghi^ |  |  | Tjempo Welut | **5.78** [±0.84]^cdefghij^ |  |  | Kamod | **1.01** [±0.03]^abcdefghij^ |  |
| Lua Chien (C 6583) | **2.45** [±0.58]^abcdefghi^ |  |  | Wase Sekitoro C | **5.88** [±0.90]^cdefghij^ |  |  | No ordem lista 18 (IC) | **1.02** [±0.15]^abcdefghij^ |  |
| **Phulpata** | **2.47** [±0.08]^abcdefghi^ |  |  | Anandi | **5.97** [±0.82]^cdefghij^ |  |  | Baek Huang Zo 59 | **1.02** [±0.01]^abcdefghij^ |  |
| **TP309** | **2.51** [±0.34]^bcdefghi^ |  |  | Ahmwe | **5.97** [±0.69]^cdefghij^ |  |  | **Shaeta** | **1.02** [±0.02]^abcdefghij^ |  |
| CR133-47 | **2.55** [±0.28]^abcdefghi^ |  |  | ARC 12771 | **5.97** [±0.56]^cdefghij^ |  |  | Saturn (Nova) | **1.15** [±0.03]^bcdefghijk^ |  |
| Hsinchu 56 | **2.64** [±0.24]^abcdefghi^ |  |  | Cau Phu Xuyen 264 | **6.04** [±0.18]^cdefghij^ |  |  | Nanhng Mon S 4 | **1.15** [±0.07]^bcdefghijk^ |  |
| **Carreon** | **2.65** [±0.77]^abcdefghi^ |  |  | Tainan-Iku 446 | **6.12** [±0.19]^cdefghijk^ |  |  | Tupa 147 | **1.16** [±0.12]^bcdefghijk^ |  |
| Tupa 147 | **2.68** [±0.61]^bcdefghi^ |  |  | Araji | **6.13** [±0.31]^cdefghijk^ |  |  | CR133-47 | **1.17** [±0.10]^cdefghijk^ |  |
| **Mapili** | **2.69** [±0.46]^bcdefghi^ |  |  | Baek Huang Zo 59 | **6.20** [±0.27]^cdefghijk^ |  |  | JW103 | **1.18** [±0.05]^cdefghijk^ |  |
| **Makapilay Pusa B** | **2.70** [±0.24]^bcdefghi^ |  |  | Saturn (Nova) | **6.22** [±0.20]^cdefghijk^ |  |  | **Hanumanjata** | **1.20** [±0.06]^defghijk^ | Above the 75^th^ percentile of vitamin B_1_ content distribution |
| Saturn (Nova) | **2.75** [±0.21]^bcdefghi^ | Above the 75^th^ percentile of vitamin B_1_ content distribution |  | E-Kha-Keha | **6.29** [±1.01]^cdefghijk^ | Above the 75^th^ percentile of vitamin B_1_ content distribution |  | **Carreon** | **1.21** [±0.21]^efghijk^ |  |
| T 1 | **2.77** [±0.28]^bcdefghi^ |  |  | Payaipatosu 4 | **6.32** [±1.12]^cdefghijk^ |  |  | **JW107** | **1.23** [±0.06]^fghijk^ |  |
| **Rei Shi Ko** | **2.87** [±0.31]^cdefghi^ |  |  | Kamod | **6.37** [±0.44]^defghijk^ |  |  | **Tapol** | **1.23** [±0.14]^ghijk^ |  |
| **Juchitan A74** | **2.87** [±0.87]^cdefghi^ |  |  | Indane | **6.54** [±0.59]^efghijk^ |  |  | **Juchitan A74** | **1.27** [±0.19]^hijk^ |  |
| Baek Huang Zo 59 | **2.94** [±0.40]^cdefghi^ |  |  | Chang Li | **6.69** [±0.65]^efghijk^ |  |  | T 1 | **1.32** [±0.17]^ijkl^ |  |
| ARC 12771 | **2.99** [±0.15]^defghi^ |  |  | **Juchitan A74** | **6.79** [±0.87]^fghijkl^ |  |  | Indane | **1.40** [±0.05]^jkl^ |  |
| **I-Kung-Pao** | **3.06** [±0.64]^efghi^ |  |  | **Fan Ho Ku** | **7.03** [±0.68]^hijkl^ |  |  | Tjempo Welut | **1.41** [±0.08]^jkl^ |  |
| Ahmwe | **3.07** [±0.60]^efghi^ |  |  | **Daw Magawk** | **7.23** [±1.24]^ghijkl^ |  |  | **Rei Shi Ko** | **1.42** [±0.12]^jkl^ |  |
| Indane | **3.11** [±0.10]^fghi^ |  |  | Tupa 147 | **7.43** [±1.33]^ijkl^ |  |  | **Phulpata** | **1.54** [±0.68]^kl^ |  |
| **Chin Ta 1-3-86** | **3.26** [±0.15]^ghi^ |  |  | **Rei Shi Ko** | **7.96** [±0.38]^jkl^ |  |  | **Aanga** | **1.55** [±0.19]^kl^ |  |
| **IR64** | **3.37** [±0.37]^hi^ |  |  | **Aanga** | **8.51** [±1.13]^kl^ |  |  | ARC 12771 | **1.57** [±0.08]^kl^ |  |
| **Tapol** | **3.68** [±0.49]^i^ |  |  | **Phulpata** | **9.15** [±1.10]^l^ |  |  | **Han Yang Zo** | **1.76** [±0.21]^l^ |  |
| Maximum fold difference | | **3.32** |  | Maximum fold difference | | **3.90** |  | Maximum fold difference | | **2.72** |

**Supplementary Table 6. Effect of polishing on vitamin B_1_ contents quantified by microbiological (yeast) assay in experiment 2.** Data presented here were used to produce Figure 2B and 2C. Accessions in bold were selected for HPLC and qRT-PCR assays. Accessions are listed in order of means for unpolished seed total vitamin B_1_ contents.

| **Accession** | **Unpolished mean**  **(ng mg DW^-1^)** | **Polished mean**  **(ng mg DW^-1^)** | **Ratio polished:unpolished** | **% loss by polishing** |  |
| --- | --- | --- | --- | --- | --- |
| Nipponbare | 3.36 | 0.65 | 5.2 | 80.7 | Below the 25^th^ percentile of vitamin B_1_ content distribution |
| **Vaid Butti** | 3.62 | 0.59 | 6.1 | 83.7 |  |
| IR 64-21 | 3.72 | 0.47 | 7.9 | 87.4 |  |
| TP309 | 3.8 | 0.5 | 7.6 | 86.8 |  |
| **Fan Ho Ku** | 3.87 | 0.61 | 6.3 | 84.2 |  |
| TAINUNG 67 | 3.9 | 0.43 | 9.1 | 89.0 |  |
| Nipponbare | 4.05 | 0.85 | 4.8 | 79.0 |  |
| **IR64** | 4.07 | 0.69 | 5.9 | 83.0 |  |
| POKKALI | 4.24 | 0.79 | 5.4 | 81.4 | Between 25^th^ and 75^th^ percentile of  vitamin B_1_ content distribution |
| Han Yang Zo | 4.25 | 1.79 | 2.4 | 57.9 |  |
| SADU CHO | 4.3 | 0.79 | 5.4 | 81.6 |  |
| JW107 | 4.4 | 0.98 | 4.5 | 77.7 |  |
| Mapili | 4.42 | 0.83 | 5.3 | 81.2 |  |
| Carreon | 4.45 | 1.06 | 4.2 | 76.2 |  |
| **I-Kung-Pao** | 4.49 | 0.72 | 6.2 | 84.0 |  |
| ZHENSHAN 97 B | 4.55 | 0.86 | 5.3 | 81.1 |  |
| Chin Ta 1-3-86 | 4.58 | 0.48 | 9.5 | 89.5 |  |
| DNJ52 | 4.67 | 0.66 | 7.1 | 85.9 |  |
| Makapilay Pusa B | 4.85 | 1.06 | 4.6 | 78.1 |  |
| **Juchitan A74** | 5.05 | 1.78 | 2.8 | 64.8 |  |
| Rei Shi Ko | 5.08 | 0.67 | 7.6 | 86.8 |  |
| **Hanumanjata** | 5.08 | 1.2 | 4.2 | 76.4 |  |
| ASWINA | 5.2 | 1.01 | 5.1 | 80.6 |  |
| **Shaeta** | 5.32 | 1.11 | 4.8 | 79.1 | Above the 75^th^ percentile of vitamin B_1_ content distribution |
| DULAR | 5.41 | 0.76 | 7.1 | 86.0 |  |
| Tapol | 5.48 | 1.66 | 3.3 | 69.7 |  |
| AZUCENA | 5.58 | 0.81 | 6.9 | 85.5 |  |
| **Aanga** | 6.15 | 1.61 | 3.8 | 73.8 |  |
| FR 13 A | 6.64 | 2.58 | 2.6 | 61.1 |  |
| **Daw Magawk** | 6.88 | 1.15 | 6.0 | 83.3 |  |
| Minimum | 3.36 | 0.43 | 2.4 | 57.9 |  |
| Maximum | 7.48 | 2.58 | 9.5 | 89.5 |  |

**Supplementary Table 7.** Experiment 1 vitamin B_6_ contents in leaves **(A)**, unpolished seeds **(B)** and polished seeds **(C)** in rice accessions grown under greenhouse conditions and quantified by microbiological (yeast) assay. The accessions with vitamin B_6_ content below the 25^th^ percentile of the distribution were considered as low vitamin B_6_ accessions and those with vitamin content above the 75^th^ percentile were considered as high vitamin B_6_ accessions. Low, intermediate and high vitamin B_6_ accessions selected for experiment 2 are bolded. Mean ± SD of 3 biological replicates, except Nipponbare (n = 6), IR64 (n = 6) and TP309 (n = 6) for the three tissues; No ordem lista 18 (IC) (n = 2) and Bansi (n = 2) for leaves; Daw Magawk (n = 2) for unpolished seeds, Daw Magawk (n = 2) and Cau Phu Xuyen 264 (n = 2) for polished seeds. Tukey's multiple comparison test (*p* < 0.05).

| **A Leaves** | | |  | **B Unpolished seeds** | | |  | **C Polished seeds** | | |
| --- | --- | --- | --- | --- | --- | --- | --- | --- | --- | --- |
| **Accession** | **Vitamin B_6_ (ng mg FW^-1^)** | |  | **Accession** | **Vitamin B_6_ (ng mg DW^-1^)** | |  | **Accession** | **Vitamin B_6_ (ng mg DW^-1^)** | |
| **Hanumanjata** | **0.88** [±0.04]^a^ | Below the 25^th^ percentile of vitamin B_6_  content distribution |  | **IR64** | **0.75** [±0.09]^a^ | Below the 25^th^ percentile of vitamin B_6_  content distribution |  | **Fan Ho Ku** | **0.34** [±0.03]^a^ | Below the 25^th^ percentile of vitamin B_6_ content distribution |
| **JW107** | **1.01** [±0.06]^ab^ |  |  | **Juchitan A74** | **0.75** [±0.07]^ab^ |  |  | LUA CHIEN (C 6583) | **0.36** [±0.03]^a^ |  |
| JW103 | **1.14** [±0.05]^ab^ |  |  | Bansi | **0.79** [±0.02]^ab^ |  |  | Nanhng Mon S 4 | **0.37** [±0.06]^ab^ |  |
| **Han Yang Zo** | **1.22** [±0.12]^ab^ |  |  | **I-Kung-Pao** | **0.80** [±0.08]^ab^ |  |  | Chang Li | **0.38** [±0.01]^ab^ |  |
| **Shaeta** | **1.36** [±0.36]^abc^ |  |  | Nanhng Mon S 4 | **0.81** [±0.01]^abc^ |  |  | **Aanga** | **0.38** [±0.01]^ab^ |  |
| Kamod | **1.40** [±0.27]^abcd^ |  |  | Ahmwe | **0.82** [±0.07]^abcd^ |  |  | Hsinchu 56 | **0.42** [±0.04]^ab^ |  |
| **Juchitan A74** | **1.42** [±0.15]^abcd^ |  |  | Araji | **0.86** [±0.04]^abcde^ |  |  | Araji | **0.44** [±0.01]^abc^ |  |
| Bansi | **1.43** [±0.37]^abcde^ |  |  | Kamod | **0.91** [±0.10]^abcde^ |  |  | Tainan-Iku 446 | **0.45** [±0.05]^abc^ |  |
| **I-Kung-Pao** | **1.45** [±0.32]^abcdef^ |  |  | **Mapili** | **0.92** [±0.06]^abcde^ |  |  | No ordem lista 18 (IC) | **0.45** [±0.02]^ab^ |  |
| **Vaid Butti** | **1.56** [±0.31]^abcdefg^ |  |  | **Hanumanjata** | **0.95** [±0.10]^abcdef^ |  |  | Gam Pai 30-12-15 | **0.45** [±0.13]^abc^ |  |
| **DNJ52** | **1.59** [±0.16]^abcdefg^ |  |  | Gam Pai 30-12-15 | **0.97** [±0.11]^abcdef^ |  |  | **Nipponbare** | **0.47** [±0.10]^abc^ |  |
| **TP309** | **1.64** [±0.68]^abcd^ |  |  | Cau Phu Xuyen 264 | **0.99** [±0.05]^abcdef^ |  |  | JW103 | **0.47** [±0.00]^abc^ | Between 25^th^ and 75^th^ percentile of vitamin B_6_ content distribution |
| **Mapili** | **1.68** [±0.45]^abcdefg^ |  |  | Hawara Batu | **1.02** [±0.00]^abcdefg^ | Between 25^th^ and 75^th^ percentile of vitamin B_6_ content distribution |  | **Chin Ta 1-3-86** | **0.47** [±0.05]^abc^ |  |
| **Makapilay Pusa B** | **1.78** [±0.28]^abcdefgh^ | Between 25^th^ and 75^th^ percentile of vitamin B_6_ content distribution |  | CR133-47 | **1.03** [±0.12]^abcdefg^ |  |  | **DNJ52** | **0.48** [±0.04]^abc^ |  |
| T 1 | **1.82** [±0.16]^abcdefgh^ |  |  | Chang Li | **1.03** [±0.09]^abcdefg^ |  |  | **I-Kung-Pao** | **0.49** [±0.10]^abc^ |  |
| Payaipatosu 4 | **1.83** [±0.39]^abcdefgh^ |  |  | No ordem lista 18 (IC) | **1.04** [±0.14]^abcdefg^ |  |  | Tjempo Welut | **0.50** [±0.07]^abc^ |  |
| **Aanga** | **1.90** [±0.45]^abcdefgh^ |  |  | **Nipponbare** | **1.06** [±0.13]^abcdef^ |  |  | Saturn (Nova) | **0.51** [±0.08]^abc^ |  |
| Wase Sekitoro C | **1.92** [±0.47]^abcdefgh^ |  |  | **Fan Ho Ku** | **1.12** [±0.10]^abcdefgh^ |  |  | Wase Sekitoro C | **0.51** [±0.09]^abc^ |  |
| **Fan Ho Ku** | **1.94** [±0.36]^abcdefghi^ |  |  | Payaipatosu 4 | **1.14** [±0.08]^abcdefghi^ |  |  | CR133-47 | **0.51** [±0.03]^abc^ |  |
| Anandi | **2.02** [±0.27]^abcdefghi^ |  |  | **Chin Ta 1-3-86** | **1.18** [±0.40]^abcdefghi^ |  |  | Indane | **0.51** [±0.06]^abc^ |  |
| Cau Phu Xuyen 264 | **2.08** [±0.64]^abcdefghi^ |  |  | Wase Sekitoro C | **1.20** [±0.20]^abcdefghi^ |  |  | **TP309** | **0.51** [±0.06]^abc^ |  |
| E-Kha-Keha | **2.13** [±0.55]^abcdefghi^ |  |  | E-Kha-Keha | **1.22** [±0.08]^abcdefghi^ |  |  | T 1 | **0.52** [±0.04]^abc^ |  |
| **Carreon** | **2.28** [±1.02]^abcdefghij^ |  |  | **TP309** | **1.22** [±0.12]^abcdefgh^ |  |  | **Shaeta** | **0.52** [±0.18]^abc^ |  |
| Chang Li | **2.33** [±0.38]^abcdefghij^ |  |  | T 1 | **1.22** [±0.07]^abcdefghi^ |  |  | **IR64** | **0.52** [±0.06]^abc^ |  |
| CR133-47 | **2.48** [±0.10]^abcdefghijk^ |  |  | Saturn (Nova) | **1.23** [±0.03]^abcdefghi^ |  |  | Kamod | **0.52** [±0.08]^abc^ |  |
| Tupa 147 | **2.49** [±0.46]^abcdefghijk^ |  |  | **Vaid Butti** | **1.23** [±0.14]^abcdefghi^ |  |  | **Makapilay Pusa B** | **0.52** [±0.06]^abc^ |  |
| Hawara Batu | **2.50** [±0.83]^abcdefghijk^ |  |  | Tung-Huan-Pe 18 | **1.26** [±0.16]^abcdefghi^ |  |  | Ahmwe | **0.53** [±0.06]^abc^ |  |
| **Phulpata** | **2.50** [±0.57]^abcdefghijk^ |  |  | Anandi | **1.26** [±0.10]^abcdefghi^ |  |  | Cau Phu Xuyen 264 | **0.53** [±0.03]^abc^ |  |
| Tung-Huan-Pe 18 | **2.53** [±0.49]^abcdefghijk^ |  |  | Lua Chien (C 6583) | **1.30** [±0.21]^abcdefghi^ |  |  | **JW107** | **0.53** [±0.05]^abc^ |  |
| Ahmwe | **2.62** [±0.13]^bcdefghijk^ |  |  | Indane | **1.33** [±0.02]^abcdefghij^ |  |  | Baek Huang Zo 59 | **0.53** [±0.01]^abc^ |  |
| Nanhng Mon S 4 | **2.89** [±0.34]^cdefghijk^ |  |  | **Shaeta** | **1.33** [±0.14]^abcdefghij^ |  |  | Hawara Batu | **0.54** [±0.04]^abc^ |  |
| **Nipponbare** | **2.94** [±1.00]^fghijk^ |  |  | Tjempo Welut | **1.40** [±0.21]^bcdefghijk^ |  |  | Tung-Huan-Pe 18 | **0.55** [±0.04]^abc^ |  |
| Hsinchu 56 | **3.01** [±0.62]^cdefghijk^ |  |  | Baek Huang Zo 59 | **1.47** [±0.09]^cdefghijkl^ |  |  | Bansi | **0.56** [±0.05]^abc^ |  |
| Tjempo Welut | **3.07** [±0.30]^efghijk^ |  |  | Hsinchu 56 | **1.49** [±0.60]^defghijkl^ |  |  | Magwak-Pi 269-7-22 | **0.56** [±0.03]^abc^ |  |
| Magwak-Pi 269-7-22 | **3.10** [±0.37]^fghijk^ |  |  | Tainan-Iku 446 | **1.50** [±0.37]^efghijklm^ |  |  | **Mapili** | **0.57** [±0.04]^abc^ | Above the 75^th^ percentile of  vitamin B_6_ content distribution |
| Lua Chien (C 6583) | **3.15** [±0.16]^ghijk^ |  |  | Tupa 147 | **1.51** [±0.10]^efghijklm^ |  |  | **Rei Shi Ko** | **0.57** [±0.05]^abc^ |  |
| Baek Huang Zo 59 | **3.20** [±0.09]^ghijk^ |  |  | Magwak-Pi 269-7-22 | **1.52** [±0.10]^efghijklm^ | Above the 75^th^ percentile of  vitamin B_6_ content distribution |  | **Hanumanjata** | **0.57** [±0.07]^abc^ |  |
| Tainan-Iku 446 | **3.21** [±0.13]^ghijk^ | Above the 75^th^ percentile of  vitamin B_6_ content distribution |  | **DNJ52** | **1.56** [±0.12]^efghijklm^ |  |  | Tupa 147 | **0.58** [±0.03]^abc^ |  |
| No ordem lista 18 (IC) | **3.25** [±0.91]^defghijk^ |  |  | **Makapilay Pusa B** | **1.60** [±0.14]^fghijklm^ |  |  | E-Kha-Keha | **0.58** [±0.10]^abc^ |  |
| **Chin Ta 1-3-86** | **3.35** [±0.67]^hijk^ |  |  | **Aanga** | **1.62** [±0.23]^fghijklm^ |  |  | Anandi | **0.60** [±0.03]^abc^ |  |
| **Rei Shi Ko** | **3.36** [±0.32]^hijk^ |  |  | **Carreon** | **1.66** [±0.12]^ghijklm^ |  |  | **Juchitan A74** | **0.60** [±0.08]^abc^ |  |
| Gam Pai 30-12-15 | **3.37** [±0.22]^hijk^ |  |  | JW103 | **1.75** [±0.03]^hijklm^ |  |  | Payaipatosu 4 | **0.60** [±0.14]^abc^ |  |
| **Daw Magawk** | **3.41** [±0.39]^hijk^ |  |  | **JW107** | **1.80** [±0.33]^ijklm^ |  |  | **Carreon** | **0.61** [±0.08]^abc^ |  |
| **IR64** | **3.43** [±0.66]^ijk^ |  |  | **Han Yang Zo** | **1.98** [±0.41]^jklmn^ |  |  | **Tapol** | **0.61** [±0.08]^abc^ |  |
| Araji | **3.91** [±0.07]^jk^ |  |  | **Rei Shi Ko** | **1.98** [±0.41]^jklmn^ |  |  | **Vaid Butti** | **0.62** [±0.01]^abc^ |  |
| **Tapol** | **3.99** [±0.94]^k^ |  |  | ARC 12771 | **2.13** [±0.13]^klmn^ |  |  | **Han Yang Zo** | **0.63** [±0.06]^abc^ |  |
| ARC 12771 | **3.99** [±0.48]^k^ |  |  | **Daw Magawk** | **2.15** [±0.04]^lmn^ |  |  | ARC 12771 | **0.66** [±0.02]^bc^ |  |
| Saturn (Nova) | **4.02** [±0.10]^k^ |  |  | **Phulpata** | **2.16** [±0.56]^mn^ |  |  | **Daw Magawk** | **0.78** [±0.11]^cd^ |  |
| Indane | **4.07** [±0.09]^k^ |  |  | **Tapol** | **2.49** [±0.38]^n^ |  |  | **Phulpata** | **1.01** [±0.42]^d^ |  |
| Maximum fold difference | | **4.64** |  | Maximum fold difference | | **3.32** |  | Maximum fold difference | | **2.97** |

**Supplementary Table 8. Effect of polishing on vitamin B_6_ contents quantified by microbiological (yeast) assay in experiment 2.** Data presented here were used to produce Figure 3B and 3C. Accessions in bold were selected for HPLC and qRT-PCR assays. Accessions are listed in order of means for unpolished seed total vitamin B_6_ contents.

| **Accession** | **Unpolished mean**  **(ng mg DW^-1^)** | **Polished mean**  **(ng mg DW^-1^)** | **Ratio**  **polished:unpolished** | **% loss**  **by polishing** |  |
| --- | --- | --- | --- | --- | --- |
| Juchitan A74 | 0.78 | 0.59 | 1.3 | 24.4 | Below the 25^th^ percentile of vitamin B_6_  content distribution |
| IR 64-21 | 0.84 | 0.58 | 1.4 | 31.0 |  |
| **Nipponbare** | 0.89 | 0.56 | 1.6 | 37.1 |  |
| Nipponbare | 0.95 | 0.56 | 1.7 | 41.1 |  |
| **IR64** | 1 | 0.6 | 1.7 | 40.0 |  |
| **I-Kung-Pao** | 1.01 | 0.65 | 1.6 | 35.6 |  |
| Chin Ta 1-3-86 | 1.07 | 0.63 | 1.7 | 41.1 |  |
| AZUCENA | 1.1 | 0.62 | 1.8 | 43.6 |  |
| **TP309** | 1.1 | 0.56 | 2.0 | 49.1 | Between 25^th^ and 75^th^ percentile of  vitamin B_6_ content distribution |
| Fan Ho Ku | 1.15 | 0.68 | 1.7 | 40.9 |  |
| TAINUNG 67 | 1.2 | 0.52 | 2.3 | 56.7 |  |
| FR 13 A | 1.23 | 0.65 | 1.9 | 47.2 |  |
| ZHENSHAN 97 B | 1.24 | 0.63 | 2.0 | 49.2 |  |
| **Aanga** | 1.24 | 0.54 | 2.3 | 56.5 |  |
| Hanumanjata | 1.25 | 0.68 | 1.8 | 45.6 |  |
| POKKALI | 1.27 | 0.6 | 2.1 | 52.8 |  |
| Mapili | 1.28 | 0.62 | 2.1 | 51.6 |  |
| JW107 | 1.28 | 0.65 | 2.0 | 49.2 |  |
| DULAR | 1.32 | 0.76 | 1.7 | 42.4 |  |
| SADU CHO | 1.36 | 0.66 | 2.1 | 51.5 |  |
| ASWINA | 1.4 | 0.76 | 1.8 | 45.7 |  |
| Rei Shi Ko | 1.4 | 0.61 | 2.3 | 56.4 |  |
| Vaid Butti | 1.42 | 0.79 | 1.8 | 44.4 |  |
| Han Yang Zo | 1.48 | 0.68 | 2.2 | 54.1 | Above the 75^th^ percentile of  vitamin B_6_ content distribution |
| **Daw Magawk** | 1.51 | 0.79 | 1.9 | 47.7 |  |
| Carreon | 1.59 | 0.64 | 2.5 | 59.7 |  |
| Makapilay Pusa B | 1.74 | 0.96 | 1.8 | 44.8 |  |
| **DNJ52** | 1.82 | 0.72 | 2.5 | 60.4 |  |
| Shaeta | 1.84 | 0.81 | 2.3 | 56.0 |  |
| **Tapol** | 3.12 | 0.81 | 3.9 | 74.0 |  |
| Minimum | 0.78 | 0.52 | 1.3 | 24.4 |  |
| Maximum | 3.12 | 0.96 | 3.9 | 74.0 |  |

**Supplementary Table 9.** Individual B_1_ vitamer profiles in leaves **(A)**, unpolished seeds **(B)** and polished seeds **(C)** in contrasting rice accessions from experiment 2, quantified by HPLC and used to construct Figure 2B–D. Data are mean ± SD of 3 biological replicates. Tukey's multiple comparison test (*p* < 0.05).

| **A** | **TDP** | | **TMP** | | **Thiamine** | | **Total vit. B_1_**  ng mg FW^-1^ |
| --- | --- | --- | --- | --- | --- | --- | --- |
| **Leaves** | ng mg FW^-1^ | % total vit. B_1_ | ng mg FW^-1^ | % total vit. B_1_ | ng mg FW^-1^ | % total vit. B_1_ |  |
| Hanumanjata | **0.99** [±0.16]^a^ | **96** [±0] | **0.03** [±0.00]^a^ | **3** [±1] | **0.02** [±0.01]^ab^ | **2** [±1] | **1.04** [±0.16]^a^ |
| Juchitan A74 | **1.02** [±0.10]^a^ | **93** [±1] | **0.02** [±0.01]^a^ | **2** [±1] | **0.05** [±0.02]^bc^ | **5** [±2] | **1.09** [±0.10]^a^ |
| Vaid Butti | **1.13** [±0.03]^a^ | **97** [±1] | **0.02** [±0.00]^a^ | **2** [±0] | **0.01** [±0.00]^a^ | **1** [±0] | **1.16** [±0.03]^a^ |
| Shaeta | **1.36** [±0.32]^ab^ | **96** [±1] | **0.03** [±0.00]^a^ | **2** [±0] | **0.02** [±0.02]^ab^ | **1** [±1] | **1.41** [±0.33]^ab^ |
| Aanga | **1.43** [±0.27]^ab^ | **95** [±1] | **0.03** [±0.01]^a^ | **2** [±0] | **0.04** [±0.00]^abc^ | **3** [±1] | **1.51** [±0.27]^ab^ |
| Daw Magawk | **1.49** [±0.06]^abc^ | **94** [±1] | **0.04** [±0.01]^a^ | **2** [±0] | **0.06** [±0.01]^c^ | **4** [±1] | **1.59** [±0.04]^abc^ |
| Fan Ho Ku | **1.79** [±0.19]^bc^ | **96** [±1] | **0.03** [±0.01]^a^ | **2** [±0] | **0.05** [±0.01]^abc^ | **3** [±1] | **1.87** [±0.18]^bc^ |
| IR64 | **1.89** [±0.21]^bc^ | **96** [±1] | **0.04** [±0.01]^a^ | **2** [±0] | **0.03** [±0.01]^abc^ | **2** [±1] | **1.97** [±0.21]^bc^ |
| I-Kung-Pao | **2.02** [±0.30]^c^ | **95** [±1] | **0.07** [±0.02]^b^ | **3** [±1] | **0.04** [±0.00]^abc^ | **2** [±0] | **2.13** [±0.32]^c^ |

| **B**  **Unpolished seeds** | **TDP** | | **TMP** | | **Thiamine** | | **Total vit. B_1_**  ng mg DW^-1^ |
| --- | --- | --- | --- | --- | --- | --- | --- |
|  | ng mg DW^-1^ | % total  vit. B_1_ | ng mg DW^-1^ | % total vit. B_1_ | ng mg DW^-1^ | % total  vit. B_1_ |  |
| IR64 | **0.05** [±0.01]^a^ | **5** [±0] | **0.01** [±0.00]^a^ | **1** [±0] | **1.00** [±0.14]^a^ | **94** [±0] | **1.07** [±0.15]^a^ |
| Vaid Butti | **0.23** [±0.09]^de^ | **18** [±3] | **0.02** [±0.01]^ab^ | **2** [±0] | **1.02** [±0.26]^a^ | **80** [±3] | **1.28** [±0.36]^a^ |
| I-Kung-Pao | **0.13** [±0.02]^abcd^ | **9** [±0] | **0.02** [±0.00]^ab^ | **2** [±0] | **1.31** [±0.13]^a^ | **90** [±0] | **1.46** [±0.14]^ab^ |
| Aanga | **0.14** [±0.02]^abcd^ | **8** [±1] | **0.02** [±0.00]^ab^ | **1** [±0] | **1.54** [±0.09]^ab^ | **90** [±1] | **1.70** [±0.11]^ab^ |
| Fan Ho Ku | **0.17** [±0.02]^bcde^ | **9** [±0] | **0.03** [±0.00]^ab^ | **1** [±0] | **1.70** [±0.11]^abc^ | **89** [±0] | **1.90** [±0.13]^abc^ |
| Hanumanjata | **0.09** [±0.01]^ab^ | **4** [±0] | **0.02** [±0.00]^ab^ | **1** [±0] | **2.08** [±0.28]^bcd^ | **95** [±0] | **2.19** [±0.30]^bcd^ |
| Juchitan A74 | **0.14** [±0.03]^abcd^ | **5** [±1] | **0.01** [±0.00]^a^ | **0** [±0] | **2.44** [±0.39]^cd^ | **94** [±1] | **2.59** [±0.41]^cd^ |
| Daw Magawk | **0.27** [±0.03]^e^ | **10** [±0] | **0.05** [±0.00]^c^ | **2** [±0] | **2.47** [±0.24]^cd^ | **88** [±0] | **2.80** [±0.27]^d^ |
| Shaeta | **0.20** [±0.04]^cde^ | **7** [±1] | **0.03** [±0.00]^ab^ | **1** [±0] | **2.67** [±0.16]^d^ | **92** [±1] | **2.90** [±0.19]^d^ |

| **C**  **Polished seeds** | **TDP** | | **TMP** | | **Thiamine** | | **Total vit. B_1_**  ng mg DW^-1^ |
| --- | --- | --- | --- | --- | --- | --- | --- |
|  | ng mg DW^-1^ | % total  vit. B_1_ | ng mg DW^-1^ | % total vit. B_1_ | ng mg DW^-1^ | % total vit. B_1_ |  |
| Hanumanjata | **0.01** [±0.00]^ab^ | **8** [±1] | **0.00** [±0.00]^ab^ | **3** [±0] | **0.09** [±0.00]^a^ | **88** [±1] | **0.10** [±0.00]^ab^ |
| I-Kung-Pao | **0.01** [±0.00]^ab^ | **10** [±0] | **0.00** [±0.00]^b^ | **3** [±1] | **0.12** [±0.01]^a^ | **87** [±1] | **0.14** [±0.01]^abc^ |
| IR64 | **0.01** [±0.00]^abc^ | **5** [±0] | **0.00** [±0.00]^ab^ | **3** [±1] | **0.15** [±0.02]^ab^ | **93** [±1] | **0.16** [±0.02]^abc^ |
| Shaeta | **0.02** [±0.01]^abc^ | **13** [±1] | **0.00** [±0.00]^b^ | **3** [±1] | **0.15** [±0.04]^ab^ | **85** [±1] | **0.17** [±0.05]^abc^ |
| Vaid Butti | **0.03** [±0.01]^c^ | **16** [±3] | **0.00** [±0.00]^a^ | **2** [±1] | **0.14** [±0.04]^a^ | **82** [±3] | **0.18** [±0.05]^abc^ |
| Aanga | **0.02** [±0.01]^bc^ | **11** [±1] | **0.00** [±0.00]^a^ | **2** [±0] | **0.18** [±0.04]^ab^ | **88** [±1] | **0.20** [±0.05]^bc^ |
| Daw Magawk | **0.03** [±0.01] | **26** [±29] | **0.00** [±0.00] | **3** [±3] | **0.19** [±0.15] | **71** [±32] | **0.22** [±0.15] |
| Fan Ho Ku | **0.02** [±0.00]^bc^ | **10** [±1] | **0.00** [±0.00]^a^ | **2** [±0] | **0.21** [±0.01]^ab^ | **89** [±1] | **0.23** [±0.01]^cd^ |
| Juchitan A74 | **0.02** [±0.01]^bc^ | **5** [±1] | **0.00** [±0.00]^a^ | **1** [±0] | **0.30** [±0.08]^b^ | **94** [±1] | **0.32** [±0.09]^d^ |

**Supplementary Table 10.** Individual B_6_ vitamer profiles in leaves **(A)**, unpolished seeds **(B)** and polished seeds **(C)** in contrasting rice accessions grown under greenhouse conditions in experiment 2 and quantified by HPLC, used to construct Figure 4B–D. Total unphosphorylated B_6_ vitamers correspond to the mean of PM, PN and PL sums for each replicate; total phosphorylated B_6_ vitamers correspond to the mean of the PMP, PNP and PLP sums for each replicate; total vitamin B_6_ corresponds to mean of total unphosphorylated + phosphorylated vitamers + PN–Glu sums for each replicate. Mean ± SD of 3 biological replicates. Tukey's multiple comparison test (*p* < 0.05). nd: not detected. Trace: B_6_ vitamer content of at least one replicate below the level of detection.

| **A** | **Leaves** | | | | | | | | | | | | |
| --- | --- | --- | --- | --- | --- | --- | --- | --- | --- | --- | --- | --- | --- |
| Vitamers  ng mg FW^-1^ | **Unphosphorylated** | | | | | **Phosphorylated** | | | | | **PN–Glu** | |  |
|  | **PM** | **PN** | **PL** | Total | % total vit. B_6_ | **PMP** | **PNP** | **PLP** | Total | % total  vit. B_6_ | **PN–Glu** | % total vit. B6 | Total vit. B_6_ |
| I-Kung-Pao | 0.38^ab^  [±0.17] | 0.23^a^  [±0.14] | 0.96^a^  [±0.10] | **1.57^a^**  [±0.39] | **65**  [±3] | 0.18^cd^  [±0.02] | 0.03^ab^  [±0.00] | 0.20^e^  [±0.03] | **0.41^c^**  [±0.05] | **17**  [±3] | **0.46^ab^**  [±0.26] | **18**  [±5] | **2.44^a^**  [±0.68] |
| DNJ52 | 0.52^abcd^  [±0.08] | 0.41^ab^  [±0.25] | 0.91^a^  [±0.28] | **1.85^ab^**  [±0.48] | **71**  [±14] | 0.16^bc^  [±0.01] | 0.03^ab^  [±0.00] | 0.18^de^  [±0.01] | **0.36^bc^**  [±0.03] | **14**  [±1] | **0.39^a^**  [±0.31] | **15**  [±12] | **2.60^ab^**  [±0.24] |
| TP309 | 0.34^a^  [±0.08] | 0.59^abc^  [±0.19] | 0.82^a^  [±0.10] | **1.75^ab^**  [±0.29] | **59**  [±5] | 0.19^cd^  [±0.01] | 0.04^ab^  [±0.02] | 0.05^abc^  [±0.02] | **0.28^b^**  [±0.04] | **10**  [±3] | **0.97^ab^**  [±0.37] | **32**  [±7] | **3.00^abc^**  [±0.60] |
| Aanga | 0.40^abc^  [±0.05] | 1.38^d^  [±0.24] | 0.73^a^  [±0.10] | **2.51^abc^**  [±0.36] | **78**  [±6] | 0.09^a^  [±0.02] | 0.02^a^  [±0.00] | trace^a^ | **0.10^a^**  [±0.02] | **3**  [±1] | **0.60^ab^**  [±0.16] | **19**  [±6] | **3.21^abc^**  [±0.24] |
| Tapol | 0.65^d^  [±0.05] | 1.21^d^  [±0.16] | 0.84^a^  [±0.09] | **2.70^bc^**  [±0.30] | **79**  [±4] | 0.13^ab^  [±0.03] | 0.03^ab^  [±0.01] | 0.01^ab^  [±0.01] | **0.17^a^**  [±0.03] | **5**  [±0] | **0.58^ab^**  [±0.20] | **16**  [±4] | **3.44^abc^**  [±0.52] |
| Nipponbare | 0.37^ab^  [±0.03] | 0.92^bcd^  [±0.23] | 0.91^a^  [±0.02] | **2.19^abc^**  [±0.23] | **59**  [±8] | 0.22^d^  [±0.01] | 0.07^b^  [±0.02] | 0.06^bc^  [±0.02] | **0.35^bc^**  [±0.02] | **9**  [±1] | **1.20^b^**  [±0.41] | **32**  [±9] | **3.74^bc^**  [±0.37] |
| IR64 | 0.59^bcd^  [±0.03] | 1.13^cd^  [±0.17] | 0.95^a^  [±0.16] | **2.66^bc^**  [±0.31] | **62**  [±5] | 0.18^cd^  [±0.01] | 0.03^ab^  [±0.00] | 0.13^d^  [±0.01] | **0.35^bc^**  [±0.01] | **8**  [±0] | **1.24b**  [±0.19] | **29**  [±5] | **4.26^cd^**  [±0.13] |
| Daw Magawk | 0.62^cd^  [±0.05] | 1.27^d^  [±0.23] | 1.00^a^  [±0.12] | **2.89^c^**  [±0.39] | **53**  [±2] | 0.18^cd^  [±0.02] | 0.05^ab^  [±0.04] | 0.07c  [±0.03] | **0.29^b^**  [±0.04] | **5**  [±0] | **2.30^c^**  [±0.29] | **42**  [±3] | **5.48^d^**  [±0.66] |

| **B** | **Unpolished seeds** | | | | | | | | | | | | |
| --- | --- | --- | --- | --- | --- | --- | --- | --- | --- | --- | --- | --- | --- |
| Vitamers | **Unphosphorylated** | | | | | **Phosphorylated** | | | | | **PN–Glu** | |  |
| ng mg DW^-1^ | **PM** | **PN** | **PL** | Total | % total vit. B_6_ | **PMP** | **PNP** | **PLP** | Total | % total vit. B_6_ | **PN–Glu** | % total vit. B_6_ | Total vit. B_6_ |
| IR64 | 0.10^a^  [±0.02] | 0.06^a^  [±0.01] | 0.06^a^  [±0.01] | **0.22^a^**  [±0.03] | **49**  [±3] | 0.02^a^  [±0.00] | nd | 0.01^abc^  [±0.00] | **0.03^a^**  [±0.00] | **7**  [±0] | **0.20^ab^**  [±0.01] | **44**  [±3] | **0.45^a^**  [±0.05] |
| Nipponbare | 0.17^ab^  [±0.01] | 0.05^a^  [±0.01] | 0.08^ab^  [±0.00] | **0.30^a^**  [±0.02] | **57**  [±0] | 0.03^ab^  [±0.00] | nd | 0.01^abc^  [±0.00] | **0.04^a^**  [±0.00] | **7**  [±1] | **0.19^ab^**  [±0.02] | **36**  [±1] | **0.53^a^**  [±0.04] |
| I-Kung-Pao | 0.21^ab^  [±0.06] | 0.07^a^  [±0.01] | 0.08^ab^  [±0.01] | **0.35^a^**  [±0.06] | **61**  [±1] | 0.04^abc^  [±0.00] | nd | trace^a^ | **0.04^ab^**  [±0.01] | **7**  [±1] | **0.18^a^**  [±0.04] | **31**  [±2] | **0.57^a^**  [±0.1] |
| TP309 | 0.17^ab^  [±0.01] | 0.09^a^  [±0.01] | 0.08^ab^  [±0.00] | **0.34^a^**  [±0.03] | **51**  [±1] | 0.02^ab^  [±0.00] | nd | trace^ab^ | **0.03^a^**  [±0.00] | **4**  [±1] | **0.30^b^**  [±0.02] | **45**  [±0] | **0.66^a^**  [±0.04] |
| Aanga | 0.38^c^  [±0.12] | 0.18^b^  [±0.02] | 0.10^b^  [±0.02] | **0.66^b^**  [±0.17] | **64**  [±7] | 0.08^e^  [±0.01] | 0.00  [±0.00] | 0.01^abc^  [±0.00] | **0.09^c^**  [±0.01] | **9**  [±0] | **0.27^ab^**  [±0.04] | **27**  [±7] | **1.01^b^**  [±0.16] |
| Daw Magawk | 0.39^c^  [±0.04] | 0.15^b^  [±0.02] | 0.09^b^  [±0.01] | **0.64^b^**  [±0.07] | **53**  [±2] | 0.06^de^  [±0.01] | 0.00  [±0.00] | 0.03^d^  [±0.01] | **0.09^c^**  [±0.01] | **8**  [±0] | **0.48^c^**  [±0.05] | **40**  [±2] | **1.21^bc^**  [±0.11] |
| DNJ52 | 0.46^c^  [±0.06] | 0.28^c^  [±0.05] | 0.06^a^  [±0.00] | **0.81^b^**  [±0.11] | **56**  [±2] | 0.04^bc^  [±0.00] | nd | 0.02^bc^  [±0.00] | **0.06^b^**  [±0.00] | **4**  [±0] | **0.58^c^**  [±0.04] | **40**  [±2] | **1.45^c^**  [±0.14] |
| Tapol | 0.32^bc^  [±0.04] | 0.26^c^  [±0.01] | 0.10^b^  [±0.00] | **0.68^b^**  [±0.04] | **39**  [±1] | 0.05^cd^  [±0.01] | nd | 0.01^bc^  [±0.00] | **0.06^b^**  [±0.01] | **3**  [±0] | **1.03^d^**  [±0.06] | **58**  [±1] | **1.78^d^**  [±0.10] |

| **C** | **Polished seeds** | | | | | | | | | | | | |
| --- | --- | --- | --- | --- | --- | --- | --- | --- | --- | --- | --- | --- | --- |
| Vitamers | **Unphosphorylated** | | | | | **Phosphorylated** | | | | | **PN–Glu** | |  |
| ng mg DW^-1^ | **PM** | **PN** | **PL** | Total | % total vit. B_6_ | **PMP** | **PNP** | **PLP** | Total | % total  vit. B_6_ | **PN–Glu** | % total vit. B_6_ | Total vit. B_6_ |
| TP309 | 0.02^a^  [±0.00] | 0.03^abc^  [±0.04] | 0.04^a^  [±0.00] | **0.10^a^**  [±0.04] | **93**  [±14] | 0.01^a^  [±0.00] | trace^a^ | nd | **0.01^a^**  [±0.00] | **7**  [±0] | **trace^a^** | **trace** | **0.10^a^**  [±0.02] |
| IR64 | 0.04^a^  [±0.00] | 0.02^ab^  [±0.01] | 0.06^a^  [±0.01] | **0.13^ab^**  [±0.00] | **91**  [±2] | 0.01^a^  [±0.00] | trace^a^ | trace^a^ | **0.01^ab^**  [±0.00] | **6**  [±2] | **0.00^a^**  [±0.00] | **3**  [±1] | **0.14^a^**  [±0.00] |
| Nipponbare | 0.07^a^  [±0.03] | 0.02^ab^  [±0.00] | 0.05^a^  [±0.00] | **0.13^ab^**  [±0.04] | **93**  [±1] | 0.01^a^  [±0.00] | trace^a^ | nd | **0.01^ab^**  [±0.00] | **7**  [±1] | **trace^a^** | **trace** | **0.14^a^**  [±0.04] |
| I-Kung-Pao | 0.08^a^  [±0.00] | 0.01^a^  [±0.01] | 0.12^a^  [±0.01] | **0.20^abc^**  [±0.02] | **85**  [±1] | 0.01^abc^  [±0.00] | 0.01^ab^  [±0.00] | trace^ab^ | **0.03^abcd^**  [±0.01] | **11**  [±1] | **0.01^a^**  [±0.00] | **4**  [±2] | **0.23^ab^**  [±0.02] |
| Aanga | 0.12^a^  [±0.10] | 0.04^abc^  [±0.01] | 0.08^a^  [±0.03] | **0.25^abc^**  [±0.15] | **87**  [±4] | 0.02^bc^  [±0.01] | 0.01^ab^  [±0.00] | nd | **0.03^cd^**  [±0.01] | **12**  [±2] | **0.02^a^**  [±0.03] | **7**  [±6] | **0.28^ab^**  [±0.16] |
| Tapol | 0.07^a^  [±0.00] | 0.08^cd^  [±0.03] | 0.11^a^  [±0.00] | **0.26^abc^**  [±0.02] | **86**  [±2] | 0.01^ab^  [±0.00] | 0.01^b^  [±0.01] | nd | **0.02^abc^**  [±0.01] | **8**  [±2] | **0.02^a^**  [±0.00] | **7**  [±1] | **0.31^ab^**  [±0.03] |
| Daw Magawk | 0.15^a^  [±0.02] | 0.07^bcd^  [±0.01] | 0.10^a^  [±0.02] | **0.32^bc^**  [±0.05] | **87**  [±1] | 0.03^c^  [±0.00] | 0.01^ab^  [±0.00] | 0.01^b^  [±0.00] | **0.04^d^**  [±0.01] | **12**  [±1] | **0.01^a^**  [±0.00] | **2**  [±1] | **0.37^b^**  [±0.06] |
| DNJ52 | 0.11^a^  [±0.01] | 0.12^d^  [±0.02] | 0.15^a^  [±0.13] | **0.38^c^**  [±0.12] | **93**  [±2] | 0.01^ab^  [±0.00] | 0.01^ab^  [±0.00] | 0.01^ab^  [±0.00] | **0.03^bcd^**  [±0.00] | **7**  [±2] | **trace^a^** | **trace** | **0.41^b^**  [±0.12] |

**Supplementary Figure 1. Conservation of RT-qPCR oligonucleotide primer binding sites in selected rice vitamin B_1_ and B_6_ biosynthesis *de novo* genes.**

** (A)** *THIC* primers bind exons 2 and 3 of the coding region (based on Nipponbare annotations). GID1 includes Nipponbare, GID30 includes I-Kung Pao, GID136 includes IR64. **(B)** *PDX1.3a* primers bind the 3ʹ UTR. GID1 comprises 1260 accessions, including 1119 *indica* accessions and includes IR64, GID2 comprises 1063 accessions, of which 884 are temperate *japonica* and includes Nipponbare, GID4 comprises 779 accessions including 254 *indica* accessions and includes I-Kung-Pao. **(C)** *PDX1.3b* binding sites. GID1 comprises 1117 accessions, including 933 *indica* accessions and includes I-Kung-Pao, GID2 comprises 956 accessions, of which 484 are *indica* and includes IR64, GID3 comprises 638 accessions including 600 temperate *japonica* and includes Nipponbare. **(D)** *PDX1.3c* primers bind the single exon in the open reading frame of the gene. GID1 comprises 1458 accessions, including 835 temperate *japonica* and includes Nipponbare. GID2 comprises 1074 accessions, of which 1361 are *indica* and includes both IR64 and I-Kung-Pao. **(E)** *PDX2* binding sites. GID1 comprises 3481 accessions, comprising 1411 *indica* accessions and includes IR64 and I-Kung-Pao, and 1134 temperate *japonica* accessions and includes Nipponbare. Forward primer binding sites are depicted by dark green boxes. Reverse primer binding sites are depicted by light green boxes and are complementary to the sequence shown. Primer sequences are listed in Supplementary Table 2.

**Supplementary Figure 2 Pearson’s correlation analyses of *PDX* expression in leaves with total vitamin B_6_ contents in unpolished seeds (A–D) and polished seeds (E–H).**

qRT-PCR data from Figure 7A–D were plotted against total vitamin B_6_ contents for unpolished seed shown in Figure 5C and polished seeds in Figure 5D.

**Supplementary Figure 3. Comparison of *A. thaliana* (Col-0) (A) and *O. sativa* (Nipponbare) (B) *THIC* promoter regions.** The CIRCADIAN CLOCK ASSOCIATED 1 binding site (AAAATATCT) is indicated in bold, underlined red font. The 5' UTR from the respective gene models is highlighted in light green, translational start codons in light blue, and an additional 1 kb of upstream sequence is shown.

**A** **At*THIC* (At2g29630), 5' UTR and 1 kb upstream of the 5' UTR.**

TAGAGAGGGGATGGTTTTATGTACGGATCGGATCGTGCGGGGAAGACAAAATAGAAAAACAACGAGGGAGTTAGTTGCTTACATGTTGTTTTTCAAAGATATTATTTTCTTCTTATTACATACACTTTTGAATTTGTTGATCGTGTTACTTACATAAAATTGCAGGTTAGGTCCCTTTGTTTTCGCAGTTTTTGCAATTATTTCTCATATTTCTTAATATTGGGCTTTTCACATGTAATAAGCCCAACGATAAGACCATGACAATTTCTATACGAAACATGATATAAATTCTTTGGATATACATTATGAATTTACGATATACAATTAGTTTGTTTAAATATCAAAATATAAATGCGTCAATGGTTGTTGTTACTTGTGAGATTATCTTTCTATTTAAGAAGAATAATTCTCTTCGTAGATAAATTTTTAAAATAATTTTCCGAGTTTTCTAATGTTTCTAGATATGATTTGATTTGAACAATTAATTCGTGGTTCTTTGAATGAATATATCGACTGTATTTGATTTCAGTTAAACTGATAATAATTGTCATTTACGTCTCAAAAGAATTGAAATATCATGTCTCTCAAGATATGGACTTACATATTGTTATGCATTATTTATCAAAATATGTGGACAAAACATAATATCAATGTCGCTTTCAGAATAATTGAACAACAGATATTGAGAAATCAATTTTTATGGTTATATCAATTGTCATTGCCAACATCTATTACATAGTAACAGTCCAATTTACATTACAATGGTAATTCAATGAAGGTAATTTACTTTTTATTGGTTTACTCGTGAAACGACGTTCTCCTCCTCACGTACCTTATCTTAATATCCTGATCAACGGACACCAATTTTCGAC**AAAATATCT**GAGAAAGAGGACACGTCAGCAAGCCTTTCGCTTTAGGCTGCATTGGGCCGTGACAATATTCAGACGATTCAGGAGGTTCGTTCCTTTTTTAAAGGACCCTAATCACTCTGAGTACCACTGACTCACTCAGTGTGCGCGATTCATTTCAAAAACGAGCCAGCCTCTTCTTCCTTCGTCTACTAGATCAGATCCAAAGCTTCCTCTTCCAGCTATG

**B** **Os*THIC* (LOC_Os03g47610), 5' UTR and 1 kb upstream of the 5' UTR.**

CGTGGGATTATATTCTTAGCACAATACCGTCCCCACCGGTACAGTATCATCCGTCATATAGCATATCAAACACTAACATACAACACCCGAGGTTCGGTAGAGGATAGCACGACAATACCACCTCAACCAACGAACAAAGGGTTTGTTCGAATTGGTGCCAAATAAACCTTATCAAAATTTGAAAATTCTAAAATTTTATAAATCGACAATATTATCAAAATTTTGATAGGATTGGCTTAATAATGTTGTCAAAATTCTATACACATTACCAATATTTAGTAACAAACTAAATATATTCATATAATTATCAATTTTACTAAAGAGTAAATTGCAATTTGGACCACCTTTTATTACCCAAGTTTCAAGTTTCAATTTGGATCACCCTTAAACATATCTTTTCAATTTGGACCGGGTAAATTTATCATTATTACGGTTTGAACTATCATGAACAACTTTTCTCGTCACGTCTAACTTCTCTTTTGGCAAACATATGAACCATACATAGAAGAGTTCGGCTGTATACTAGAGCTGATGTGCTTAAGGTCGTTGTTTGTACTGAAAAAATCGTTCGTGGTGGTCCAAATTATAACAATAGCAAATTTATCCGGTTCAAAGTGAAAAGATATGTTTAAGGGTGGTCCAAAATAAAACTTAGGTAATAAAATCTGGCCCAAAACGTAATTCAATCTTTACTAAATAATTGTATGGCTGAAAACGTCATCAATAAACAGGCCCAAAATTAACCGTCCCCATACTACTGCAAACGCCACGCCATCATCCCTCCTGTCCTAATCAATTCTCTTTTTCGGACCGTTGGAAGCGTCCGCTGGCGCAGAGAAAGTATCTGTGGTGGCCGGACAGAAATATCTCCCCTCCCGTCGGCCGTCGCTTTTGGCCGTCCGTTCTCTCTTTTGGCCACCCGTCCGCTATCCTCCCCCCAAATCTGCAATCCCCCGCGCGCCCCCCGCTATA**AAAATATCT**TCCGCCCTTGCACCCCTCCCCCCCATCCCGCTCACGAGTTCCTCACTTCCTCCTCCGCCCGCGTCGCCCCTCCTCTCCTCTCCCTCCCGGCTCCCGGCTACCGCCGCCGCCGCCCCGAGGAAATG

**References**

Dell'Aglio E, Boycheva S, Fitzpatrick TB (2017) The pseudoenzyme PDX1.2 sustains vitamin B6 biosynthesis as a function of heat stress. *Plant Physiology* 174: 2098-2112

Jain M, Nijhawan A, Tyagi AK, Khurana JP (2006) Validation of housekeeping genes as internal control for studying gene expression in rice by quantitative real-time PCR. *Biochemical and Biophysical Research Communications* 345: 646-651

Kennedy G, Burlingame B (2003) Analysis of food composition data on rice from a plant genetic resoruces perspective. *Food Chemistry* 80: 589-596

McNally KL, Childs KL, Bohnert R, Davidson RM, Zhao K, Ulat VJ, Zeller G, Clark RM, Hoen DR, Bureau TE *et al* (2009) Genomewide SNP variation reveals relationships among landraces and modern varieties of rice. *Proceedings of the National Academy of Sciences* 106: 12273-12278

Sotelo A, Sousa V, Montalvo I, Hernandez M, Hernandez-Aragon (1990) Chemical composition of different fractions of 12 Mexican varieties of rice obtained during milling. *Cereal Chemistry* 67: 209-212

Villareal CP, Juliano BO (1989) Variability in contents of thiamine and riboflavin in brown rice, crude oil in brown rice and bran-polish, and silicon in hull of IR rices. *Plant Foods for Human Nutrition* 39: 287-297
